# Comparison of Anti-Acute Phase Effect of CIGB-258 and Its Wild-Type Peptide (E18-3) in a Hyperinflammatory and Acute Bleeding Model of Zebrafish: A Surface Plasmon Resonance Study to Compare Binding Affinity with High-Density Lipoproteins

**DOI:** 10.3390/ijms27104516

**Published:** 2026-05-18

**Authors:** Kyung-Hyun Cho, Yunki Lee, Sang Hyuk Lee, Ashutosh Bahuguna, Seung Hee Baek, María del Carmen Domínguez-Horta, Gillian Martínez-Donato

**Affiliations:** 1 Raydel HDL Research Institute, Medical Innovation Complex, Daegu 41061, Republic of Korea; 2 Center for Genetic Engineering and Biotechnology, Ave 31, e/158 y 190, Playa, Havana 10600, Cuba

**Keywords:** binding affinity, carboxymethyllysine, CIGB-258 (Jusvinza^®^), ethanol, high-density lipoproteins, surface plasmon resonance (SPR), zebrafish

## Abstract

The study compares the effects of the HSP60-derived mutated peptide (CIGB-258) and its wild-type peptide (E18-3) on preventing carboxymethyllysine (CML)- and ethanol (Et-OH)-induced hemorrhagic events and acute toxicity in zebrafish. The results suggest a 67% survivability and swimming recovery in CIGB-258-treated zebrafish compared to only 20% in the CML+Et-OH-treated group. No effect of E18-3 was noticed on CML+Et-OH-impaired zebrafish survivability and swimming ability. Similarly, no effect of E18-3 was noticed on the CML+Et-OH-disturbed blood oxidative and antioxidant variables. In contrast, CIGB-258 showed a notable 35% lower rate of oxidized contents, and 2.0-fold and 1.2-fold higher paraoxonase (PON) and ferric ion reduction activity (FRA), respectively, than in the E18-3 group. Also, the CML+Et-OH-induced dyslipidemia was substantially prevented by the CIGB-258, whereas no protective effect of E18-3 was noticed. Similarly, the CML+Et-OH-triggered hepatic inflammation, steatosis, kidney damage, severe gastrointestinal bleeding, and intestinal fibrosis were successfully mitigated by co-treatment with CIGB-258. Surface plasmon resonance analysis revealed a substantial binding affinity of CIGB-258 for HDL_2_ and HDL_3_, characterized by association rate constants (K_a_) of 14.78 and 6.20 μM^−1^s^−1^, dissociation rate constants (K_d_) of 0.35 s^−1^ and 0.22 s^−1^, and equilibrium dissociation constants (*K_D_*) of 0.024 and 0.035 μM, respectively. In conclusion, CIGB-258 exerted a substantial impact on CML+Et-OH-triggered adverse events, with high affinity for HDL, whereas E18-3 exposure remained unaffected and failed to produce any beneficial effects.

## 1. Introduction

Hyperinflammation in response to a variety of stimuli causes multiple organ damage, leading to the life-threatening condition of septic shock [1]. Acute hyperinflammation and many infectious diseases are often associated with a phenomenon known as a cytokine storm, which is characterized by an excessive release of inflammatory cytokines, such as tumor necrosis factor (TNF)-α and interleukin (IL)-6, due to activation of the immune system [2]. Bacterial endotoxins (lipopolysaccharides) and advanced glycation end products (AGEs) like carboxymethyllysine (CML) activate the immune system by interacting with toll-like receptor (TLR)-4 and receptors for advanced glycation end products (RAGEs) [3,4], leading to the production of various chemokines and cytokines.

AGEs are harmful compounds that primarily target proteins but can also affect DNA and lipids, leading to functional impairment of tissues and organs that play important roles in various diseases [5]. Accumulation of AGE hinders normal cellular signalling, and its binding with RAGEs activates several signaling events, including activation of nuclear factor kappa-light-chain-enhancer of activated B cell (NFκB) pathway, resulting in the production of cytokines like TNF-α, IL-6 and IL-1β that provide a conducive environment for the development of various diseases [6]. In addition, AGEs (like CML) also exert epigenetic effects by inducing demethylation of the RAGE promoter, thereby enhancing RAGE expression [7]. The subsequent interaction between overexpressed RAGEs and AGEs triggers a range of adverse cellular and pathological events.

Heat shock proteins (HSPs) are a group of proteins that are immunomodulatory and are typically induced by stress, playing an important role in cryoprotection [8]. For instance, HSP60 has several epitopes that induce inflammation, while the others are immunoregulatory [9]. From HSP60, a 27-amino-acid peptide, E18-3 was derived using the amino acid sequence 83–109 [10]. Further, a point mutation on E18-3 was induced at position 18 by replacing aspartic acid with leucine to develop an altered peptide ligand (APL) that was later named CIGB-258 (Jusvinza^®^) [9,10]. This mutation conferred CIGB-258, which has a higher affinity for human leukocyte antigen (HLA)-II than the wild-type E18-3 [9,10], thereby imparting a strong immunomodulatory role to CIGB-258. Some earlier comparative studies showed a positive effect of CIGB-258 on the proliferation of immunosuppressive regulatory T cells (T_reg_) and a reduction in TNF-α, while no effect of E18-3 was reported [11]. Further clinical studies in patients with rheumatoid arthritis (RA) revealed that CIGB-258 inhibits proinflammatory IL-17 and promotes T_reg_ cell proliferation, thereby balancing the physiological ratio of IL-17/T_reg_ cells [9]. In patients with COVID-19, CIGB-258 was reported to have a substantial effect, preventing hyperinflammation by inhibiting cytokine efflux and simultaneously activating T_reg_ cells [12]. In addition to the immunomodulatory role, CIGB-258 has been documented to stabilize the high-density lipoprotein (HDL) structure [13,14,15] and to augment HDL functionality [16].

Although CIGB-258 has demonstrated significant therapeutic potential in multiple inflammatory conditions [9,12], its comparative efficacy with the wild-type peptide (E18-3) in sepsis-like hyperinflammatory models remains poorly explored. In view of this, the present study was conducted to examine the comparative effects of CIGB-258 and E18-3 on CML+ethanol (Et-OH)-induced acute mortality, metabolic disturbances, and organ damage in adult zebrafish (*Danio rerio*). In addition, the binding affinities of CIGB-258 and E18-3 for HDL were determined using a surface plasmon resonance (SPR) approach.

Zebrafish was chosen as a model organism owing to its substantial similarity to many pathways in human diseases [17], along with its high physiological and genetic resemblance to humans [18]. The zebrafish genome has been completely sequenced [19], and comparative genomic studies have revealed that about 70–82% of important inflammatory pathways are conserved between zebrafish and humans, including NFκB and NLRP3 signaling pathways [20]. Exposure of external stimuli such as LPS activates the TLR-4/NFκB signaling pathway in zebrafish and increases the expression of pro-inflammatory cytokines [20,21], mimicking human inflammatory responses. Also, the production of various cytokines and the immune response of zebrafish closely match those of mice in many respects [22]. However, easy husbandry and the small size of zebrafish (around 3~4 cm) [23] make it possible to recruit a large number of participants in a limited space, which is relatively convenient compared to the other rodent models. Due to the aforementioned properties, zebrafish are gaining substantial attention as a reliable animal model for studying sepsis [22,24] and inflammatory disorders [17,18]. Thus, findings from zebrafish can provide useful information for designing a study in human subjects.

## 2. Results

### 2.1. Survivability and Swimming Ability

**Figure 1:**
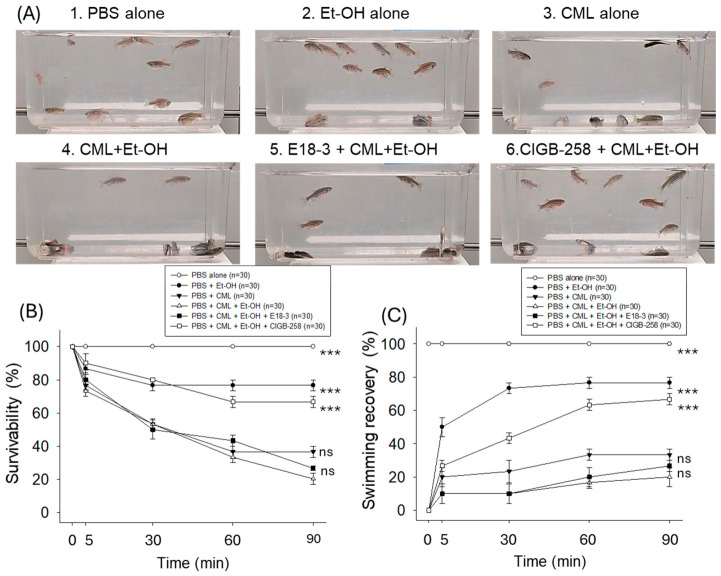
Comparative effects of peptide E18-3 and CIGB-258 on the survivability and swimming behavior of zebrafish exposed to ethanol (Et-OH), carboxymethyllysine (CML), and CML+Et-OH. (**A**) Still images depicting the swimming activity at 90 min post-treatment. (**B**,**C**) Time-dependent (0–90 min) survivability and swimming recovery, respectively, across the different treated groups. Statistical difference vs. CML+Et-OH is marked by *** at *p* < 0.001; ns: non-significant difference.

As shown in Figure 1A and Supplementary Videos S1–S6, the CML+Et-OH group showed the least survivability, around 20% at 90 min post-treatment, while the PBS (control) group showed 100% survivability. The Et-OH-only group and the CML-only group showed 76% and 37% survivability, respectively, against only 20% survivability in the CML+Et-OH group (Figure 1B). The co-treatment of CIGB-258 with CML+Et-OH resulted in increased survivability of around 67%, while the co-treatment of E18-3 resulted in lower survivability of around 26%, which is statistically similar to the survivability observed in the CML+Et-OH group.

As shown in Figure 1C, the CML+Et-OH group showed the lowest swimming recovery of zebrafish, around 20% at 90 min post-treatment, while the PBS (control) group showed 100% recovery. The Et-OH-only group and the CML-only group showed 76% and 33% swimming recovery, respectively, while the combination (CML+Et-OH) showed 20% recovery. The co-treatment of CIGB-258 with CML+Et-OH resulted in an increase in swimming ability of around 67%, while co-treatment of E18-3 resulted in much lower swimming recovery, of around 26%, which was statistically similar to the recovery rate of the CML+Et-OH group.

No significant differences in body weight were observed between baseline measurements (before treatment) and body weight recorded at 30–180 min post-treatment in any of the groups.

### 2.2. Oxidative Status and Antioxidant Activities in Zebrafish Plasma

**Figure 2:**
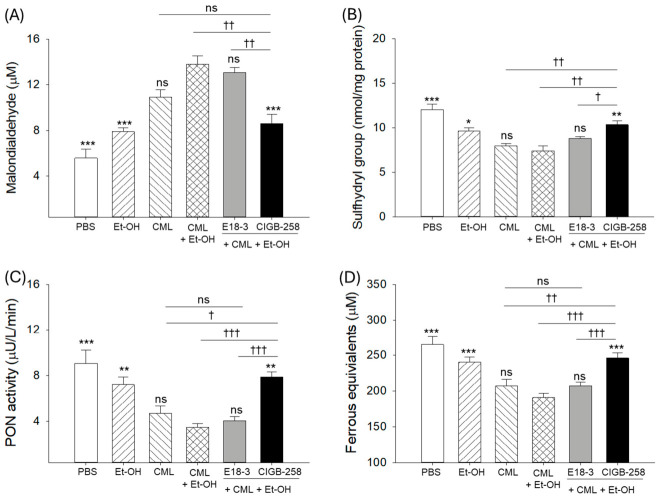
Comparative effects of peptide E18-3 and CIGB-258 intraperitoneal injections on blood (**A**) malondialdehyde (MDA), (**B**) sulfhydryl content, (**C**) paraoxonase (PON) activity, and (**D**) ferric ion reduction ability (FRA) of zebrafish exposed to ethanol (Et-OH), carboxymethyllysine (CML), and CML+Et-OH. All analyses were performed 180 min after treatment. Statistical difference vs. CML+Et-OH group is marked by * (*p* < 0.05), ** (*p* < 0.01), and *** (*p* < 0.001). The symbols highlighted by ^†^ (*p* < 0.05), ^††^ (*p* < 0.01), and ^†††^ (*p* < 0.001) represent the statistical difference between the marked groups; ns: non-significant difference.

As shown in Figure 2A, quantification of oxidized species in plasma by thiobarbituric acid reactive substance assay using malondialdehyde (MDA) revealed that the CML+Et-OH group showed the highest oxidative species, 2.5-fold (*p* < 0.001) higher than the PBS (control) group. In contrast, the Et-OH-only and CML-only groups showed only 1.4- and 2.0-fold higher MDA levels, respectively, than the PBS-only group. Co-treatment of CIGB-258 with CML+Et-OH resulted in a significantly lower MDA level (1.6-fold, *p* < 0.001) compared to the CML+Et-OH group. In contrast to this, the treatment of E18-3 did not cause any substantial reduction in the CML+Et-OH group’s elevated MDA level.

As shown in Figure 2B, the PBS-only group showed the highest sulfhydryl (SH) content, around 12 nmol/mg of protein, while the CML+Et-OH group showed the lowest SH content, around 1.6-fold (*p* < 0.001) lower than the PBS group. The individual treatment of Et-OH only and CML only displayed a lower effect than the combination (CML+Et-OH) on decreasing plasma SH content. Treatment of CIGB-258 resulted in a significant increase in SH content, around 1.4-fold (*p* < 0.01) higher than in the CML+Et-OH group, while the co-treatment of E18-3 did not significantly increase the CML+Et-OH-diminished SH content.

As shown in Figure 2C,D, the PBS-only group showed the highest paraoxonase (PON) activity (9.1 ± 1.2 μU/L/min) and ferric ion reduction ability (FRA) (265.1 ± 11.1 μM), which were significantly decreased in the CML+Et-OH-treated group (2.5-fold, *p* < 0.001; and 1.4-fold, *p* < 0.001). The E18-3 co-treatment with the CML+Et-OH group showed no effect on the elevation in PON and FRA, whereas the CIGB-258 group showed significantly enhanced FRA and PON activities. Notably, compared to the E18-3 group, significant increases in PON and FRA activity (1.9-fold, *p* < 0.001; and 1.2-fold, *p* < 0.001) were observed in the CIGB-258-treated group. The findings underscore that the CML+Et-OH-induced elevation in oxidative stress and decrease in plasma antioxidant activities were significantly improved by exposure to CIGB-258, but not to E18-3.

### 2.3. Lipid Profiles in the Zebrafish Plasma

**Figure 3:**
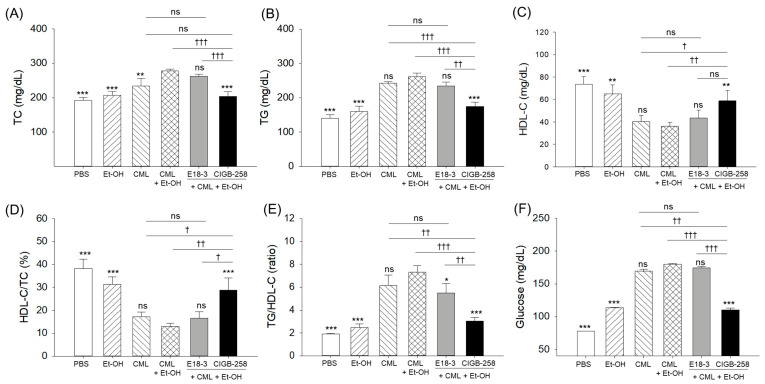
Comparative effects of peptide E18-3 and CIGB-258 treatments on blood (**A**) total cholesterol (TC), (**B**) triglycerides (TG), (**C**) high-density lipoprotein cholesterol (HDL-C), (**D**) percentage ratio of HDL-C/TC, (**E**) TG/HDL-C ratio, and (**F**) glucose levels of zebrafish exposed to ethanol (Et-OH), carboxymethyllysine (CML), and CML+Et-OH. All analyses were performed 180 min after intraperitoneal injection. Statistical difference vs. CML+Et-OH group is marked by * (*p* < 0.05), ** (*p* < 0.01), and *** (*p* < 0.001). The symbols highlighted by ^†^ (*p* < 0.05), ^††^ (*p* < 0.01), and ^†††^ (*p* < 0.001) represent the statistical difference between the marked groups; ns: non-significant difference.

As shown in Figure 3A–C, the CML+Et-OH group showed the highest TC and TG levels and the lowest HDL-C levels, which were around 1.4-fold (*p* < 0.001) and 1.9-fold (*p* < 0.001) higher and 2.1-fold (*p* < 0.001) lower than the respective levels observed in the PBS (control) group. The treatment of CIGB-258 showed a 1.4-fold (*p* < 0.001) and 1.5-fold (*p* < 0.001) reduction in TC and TG levels, and a 1.6-fold (*p* < 0.01) elevation in HDL-C levels compared to the respective levels in the CML+Et-OH group. In contrast, no significant effect of E18-3 was noticed on the reduction in TC and TG levels or the elevation in HDL-C level imparted by exposure to CML+Et-OH.

Likewise, the lowest HDL-C/TC (%) and the highest TG/HDL-C ratios were observed in the CML+Et-OH group, which were significantly lower (2.9-fold, *p* < 0.001) and higher (3.8-fold, *p* < 0.001) than the respective ratios in the PBS group (Figure 3D,E). The CML+Et-OH-disturbed HDL-C/TC (%) and TG/HDL-C ratios were substantially reverted by the co-treatment of CIGB-258. Unlike CIGB-258, no effect of E18-3 was observed on the CML+Et-OH-disturbed HDL-C/TC (%) and TG/HDL-C ratios.

Consistently, a 2.3-fold (*p* < 0.001) increase in plasma glucose was observed in the CML+Et-OH group compared to the PBS (control) group (Figure 3F). The CIGB-258-treated group showed significantly reduced (1.6-fold, *p* < 0.001) blood glucose levels compared to the CML+Et-OH group. No significant effect of E18-3 treatment was observed on the CML+Et-OH-elevated plasma glucose level.

### 2.4. Histological Analysis of Hepatic Tissue

**Figure 4:**
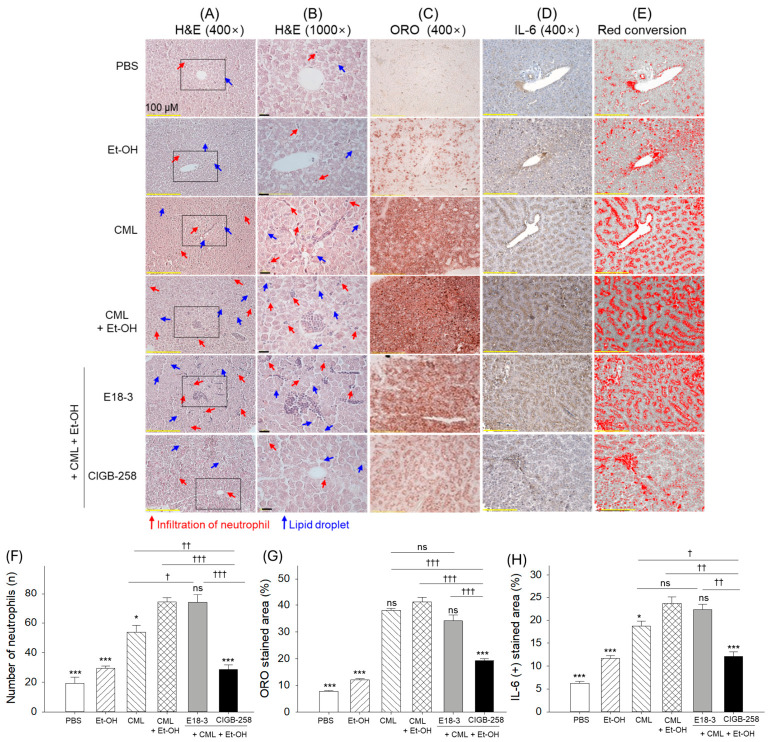
A comparative histological analysis of zebrafish liver 180 min after intraperitoneal injection of E18-3 and CIGB-258 in the presence of carboxymethyllysine (CML) and ethanol (Et-OH). (**A**,**B**) The images from the hematoxylin and eosin (H&E) staining captured at 400× and 1000× magnification, respectively. Red arrow and blue arrow highlights neutrophils and lipid droplets, respectively. The area within the black box is shown as 1000× magnifiaction in image B. (**C**) Images from the oil red O staining. (**D**) Immunohistochemical (IHC) images depicting interleukin (IL)-6. (**E**) IHC-stained area (brown color) was converted to red; red conversion was performed at a brown color threshold value (20–120) using ImageJ software (https://imagej.net/ij, version 153, assessed on 17 June 2025) to enhance visibility. (**F**–**H**) Quantification of neutrophils, ORO and IL-6-stained area, receptively. Statistical difference vs. CML+Et-OH group is marked by * (*p* < 0.05) and *** (*p* < 0.001). The symbols highlighted by ^†^ (*p* < 0.05), ^††^ (*p* < 0.01), and ^†††^ (*p* < 0.001) represent the statistical difference between the marked groups; ns: non-significant difference.

As shown in Figure 4A–C, the CML+Et-OH group showed the highest extent of inflammatory tissue morphology, with the appearance of heightened neutrophil infiltrations and lipid droplets, as denoted by the red arrow and blue arrow, respectively. The quantitative outcomes from H&E and ORO staining revealed significantly higher numbers for neutrophil (3.9-fold, *p* < 0.001) and lipid accumulation (5.2-fold, *p* < 0.001), respectively) in the CML+Et-OH group compared to the PBS (control) group (Figure 4F,G). No effect of E18-3 treatment was noticed to prevent the CML+Et-OH-triggered neutrophil infiltrations and lipid droplets. However, CIGB-258 treatment showed substantial inhibition of CML+Et-OH inflammation and fatty liver changes, reflected by 2.6-fold (*p* < 0.001) and 2.1-fold (*p* < 0.001) lower neutrophil counts and lipid accumulation, respectively, compared to the CML+Et-OH group.

Immunohistochemistry analysis revealed that the CML+Et-OH group showed the highest IL-6 positive-stained area, which was significantly higher (3.8-fold, *p* < 0.001) than the IL-6-stained area in the PBS group (Figure 4D,E,H). The E18-3-treated group showed a similar level of IL-6 expression to the CML+Et-OH group; however, the CIGB-258-treated group showed a 1.9-fold (*p* < 0.001) lower IL-6-stained area than the CML+Et-OH group.

### 2.5. Oxidative Stress and Cellular Senescence in Hepatic Tissue

**Figure 5:**
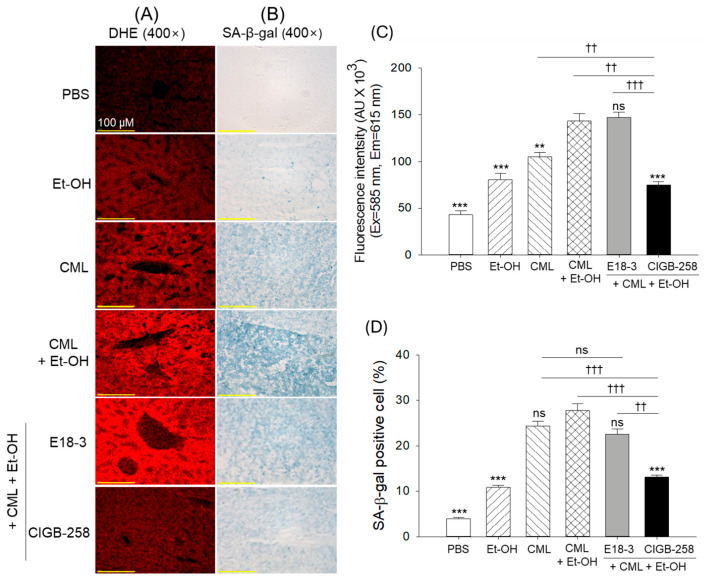
Comparative effects of intraperitoneal injection of E18-3 and CIGB-258 in the presence of carboxymethyllysine (CML) and ethanol (Et-OH) on zebrafish liver reactive oxygen species (ROS) production and senescence. (**A**) Dihydroethidium (DHE) fluorescent staining for the detection of ROS. (**B**) Senescence-associated β-galactosidase (SA-β-gal) staining to detect cellular senescence. (**C**,**D**) Quantification of the DHE fluorescent intensity and senescent-positive cells, respectively. Statistical difference vs. CML+Et-OH group is marked by ** (*p* < 0.01) and *** (*p* < 0.001). The symbols highlighted by ^††^ (*p* < 0.01) and ^†††^ (*p* < 0.001) represent the statistical difference between the marked groups; ns: non-significant difference.

As shown in Figure 5A–D, the CML+Et-OH group showed the strongest DHE fluorescent intensity (red fluorescence to represent ROS production) and cellular senescence, while the PBS-only group showed the weakest red fluorescence and senescence, which were significantly lower (3.3-fold, *p* < 0.001; and 7.1-fold, *p* < 0.001) than in the CML+Et-OH group. The CIGB-258-treated group showed significantly lower DHE-fluorescence intensity and cellular senescence, with levels 1.9-fold (*p* < 0.001) and 2.1-fold (*p* < 0.001) lower, respectively, than those of the CML+Et-OH group. No significant effect of E18-3 treatment was observed on the elevated DHE fluorescence and cellular senescence in response to CML+Et-OH.

### 2.6. Hepatic Enzyme Levels in the Zebrafish Plasma

**Figure 6:**
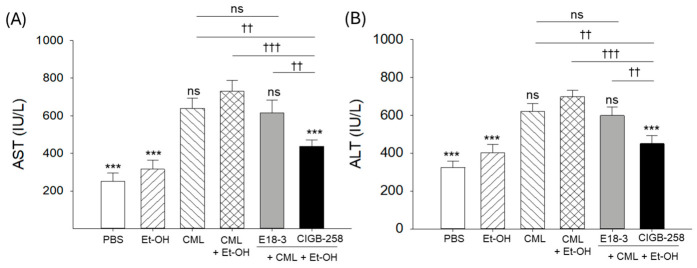
Comparative levels of zebrafish blood hepatic function biomarkers (**A**) aspartate aminotransferase (AST) and (**B**) alanine aminotransferase (ALT) 180 min after intraperitoneal injection of E18-3 and CIGB-258 in the presence of carboxymethyllysine (CML) and ethanol (Et-OH). Statistical difference vs. CML+Et-OH group is marked by *** (*p* < 0.001). The symbols highlighted by ^††^ (*p* < 0.01) and ^†††^ (*p* < 0.001) represent the statistical difference between the marked groups; ns: non-significant difference.

As shown in Figure 6, the CML+Et-OH group showed the highest AST and ALT levels, which were significantly higher (2.8-fold, *p* < 0.001; and 2.2-fold, *p* < 0.001) than the respective levels quantified in the PBS (control) group. Compared to the CML+Et-OH group, the CIGB-258-treated group showed notably lower AST and ALT levels, at 1.7-fold (*p* < 0.001) and 1.5-fold (*p* < 0.001), respectively. In contrast, no effect of E18-3 treatment was observed on the CML+Et-OH-elevated AST and ALT levels.

### 2.7. Kidney Histology

**Figure 7:**
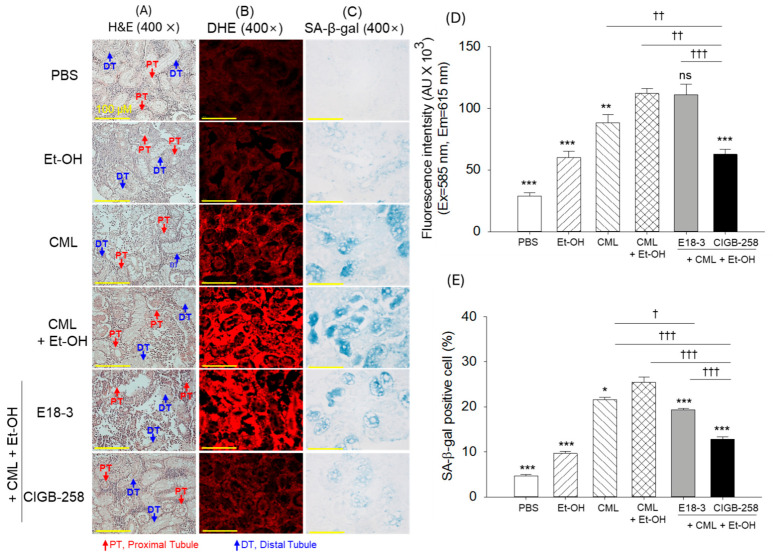
A comparative histological analysis of zebrafish kidney 180 min after intraperitoneal injection of E18-3 and CIGB-258 in the presence of carboxymethyllysine (CML) and ethanol (Et-OH). (**A**) The images from the hematoxylin and eosin (H&E) staining were captured at 400× magnification. Red and blue arrows highlight proximal tubules and distal tubules, respectively. (**B**) Images from dihydroethidium (DHE) fluorescent staining. (**C**) Senescence-associated β-galactosidase (SA-β-gal) staining. (**D**,**E**) Quantification of DHE fluorescent intensity and senescent positive cells, respectively. Statistical difference vs. CML+Et-OH group is marked by * (*p* < 0.05), ** (*p* < 0.01) and *** (*p* < 0.001). The symbols highlighted by ^†^ (*p* < 0.05), ^††^ (*p* < 0.01), and ^†††^ (*p* < 0.001) represent the statistical difference between the marked groups; ns: non-significant difference.

As shown in Figure 7A, H&E staining revealed highly dense and organized proximal tubules (PTs, highlighted by red arrow) and distal tubules (DTs, highlighted by blue arrow) in the kidney section of the PBS-treated group. In contrast to this, substantial changes in the tubular structures and arrangement appeared in the Et-OH- and CML-treated groups. However, the combined supplementation of CML+Et-OH displayed a more severe effect, as reflected by the highly distorted and sparsely populated tubular structures. The CIGB-258 group showed effective inhibition of the CML+Et-OH-induced histological changes in the kidney and preserved kidney cellular integrity, while the E18-3 group did not show a protective effect.

The DHE and SA-β-gal staining showed the highest red (DHE) fluorescence intensity and the greatest number of blue-stained (SA-β-gal positive) cells, corresponding to ROS generation and cellular senescence in the kidney of the CML+Et-OH-treated group. These levels were 3.8-fold (*p* < 0.001) and 5.9-fold (*p* < 0.001) higher than their corresponding basal levels in the PBS (control) group (Figure 7B–E). The treatment of CIGB-258 protected the kidney from CML+Et-OH-induced ROS generation and cellular senescence, reflected by the significantly lower DHE fluorescence and SA-β-gal positive cells, at 1.8-fold (*p* < 0.001) and 1.9-fold (*p* < 0.001), respectively, than the CML+Et-OH group. Contrary to this, no significant effect of E18-3 was observed on the CML+Et-OH-induced DHE fluorescence or cellular senescence.

### 2.8. Gastrointestinal Bleeding and Clotting

**Figure 8:**
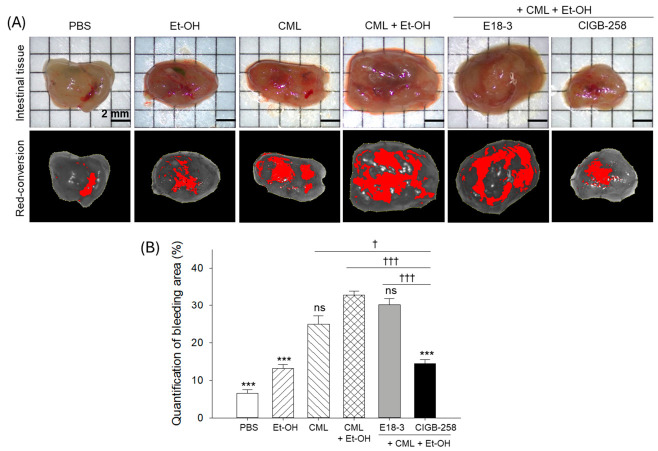
Comparative effects of CIGB-258 and E18-3 on zebrafish (**A**) intestinal bleeding at 180 min post-intraperitoneal injection in the presence of CML and Et-OH. Red conversion of the bleeding area was performed using the Image J platform (https://image.net/ij, version 1.53, accessed on 17 June 2025) with a red color threshold of 20–120 to improve the visibility of the bleeding area. (**B**) Quantification of the intestinal bleeding area. Statistical difference vs. CML+Et-OH group is marked by *** (*p* < 0.001). The symbols highlighted by ^†^ (*p* < 0.05) and ^†††^ (*p* < 0.001) represent the statistical difference between the marked groups; ns: non-significant difference.

As depicted in Figure 8, the CML+Et-OH group showed the highest intestinal bleeding, which was significantly higher (4.8-fold, *p* < 0.001) than the bleeding noticed in the PBS group. The individual treatment of ethanol had no effect on intestinal bleeding compared with the PBS group. CML alone substantially enhanced the intestinal bleeding; however, CML in combination with Et-OH (CML+Et-OH) elevated bleeding maximally. CIGB-258 treatment effectively reduced intestinal bleeding, as evidenced by the 2.3-fold (*p* < 0.001) reduction in bleeding area compared to the CML+Et-OH group. Unlike CIGB-258, no effect of E18-3 was observed to prevent intestinal bleeding augmented by the exposure to CML+Et-OH.

### 2.9. Histology of Intestine

**Figure 9:**
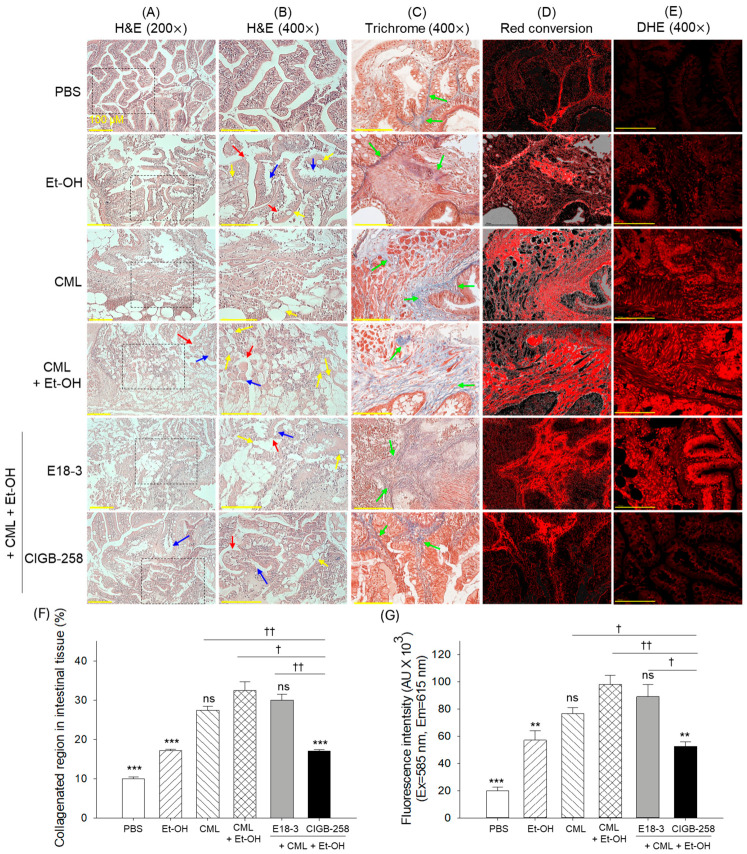
A comparative intestinal histological analysis of zebrafish 180 min after intraperitoneal injection of E18-3 and CIGB-258 in the presence of carboxymethyllysine (CML) and ethanol (Et-OH). (**A**,**B**) The images from the hematoxylin and eosin (H&E) staining captured at 200× and 400× magnification, respectively. The area within the black box is shown as 400× magnification in image B Red, blue, and yellow arrows depict the enteric villus dissolution, degeneration of lamina propria, and shrinkage and swelling of the goblet cells, respectively. (**C**) Images from Masson-trichrome-stained intestinal microsection. Green arrow highlights collagenated region. (**D**) Red conversion of the blue-stained collagen, as seen in the Masson-trichrome staining, was performed to enhance visibility at a blue color threshold of 20–120 using ImageJ (https://imagej.net/ij, version 1.53, assessed on 18 July 2025). (**E**) A 400× magnified view of the red conversion images depicted in subfigure D. (**F**,**G**) Quantification of the collagenated region and DHE fluorescent intensities. Statistical difference vs. CML+Et-OH group is marked by ** (*p* < 0.01) and *** (*p* < 0.001). The symbols highlighted by ^†^ (*p* < 0.05) and ^††^ (*p* < 0.01) represent the statistical difference between the marked groups; ns: non-significant difference.

The H&E staining depicted a well-organized villi structure with intact lamina propria in the PBS-treated group (Figure 9A,B). Individual treatment of Et-OH and CML had a substantial adverse effect on the intestinal histology, reflected by the enteric villus dissolution (highlighted by the red arrow) and degeneration of lamina propria (highlighted by the blue arrow). In addition, shrinkage and swelling of the goblet cells (highlighted by the yellow arrow) were also noticed. Contrary to the individual treatment, a combined treatment of CML+Et-OH led to a more severe effect on villus integrity and lamina propria degeneration. The exposure of E18-3 displayed no protective effect on the CML+Et-OH-triggered intestinal damage. In contrast, CIGB-258 treatment substantially prevented intestinal damage caused by the CML+Et-OH. Nevertheless, disrupted villus architecture and lamina propria, along with goblet cell hypertrophy, were observed at some sites.

The Masson-trichrome staining of the intestinal tissue showed a significant elevation in the collagenated region in the CML+Et-OH group, which was significantly higher than the collagenated region (highlighted by the green arrow) of the PBS- and Et-OH-treated groups, at 3.2-fold (*p* < 0.001) vs. 1.9-fold (*p* < 0.001) higher, respectively (Figure 9C,D,F). The treatment of E18-3 had no substantial effect against CML+Et-OH-induced collagenation. In contrast, CIGB-258 treatment effectively mitigated CML+Et-OH-induced disruption of the inner circular muscle and inhibition of tissue fibrosis, as reflected by the 1.9-fold (*p* < 0.001) reduction in the collagenated region compared to the CML+Et-OH group.

The DHE staining revealed the highest DHE fluorescence in the CML+Et-OH-treated group, which was significantly4.9-fold (*p* < 0.001) and 1.7-fold (*p* < 0.01) higher than the DHE fluorescence detected in the PBS- treated and Et-OH-treated groups, respectively. No effect of E18-3 treatment was observed to reduce the CML+Et-OH-heightened DHE fluorescence (Figure 9E,G). In contrast, the treatment of CIGB-258 substantially reduced DHE fluorescence by 1.9-fold (*p* < 0.01) compared to the CML+Et-OH group, attesting to CIGB-258’s preventive effect on CML+Et-OH-induced ROS generation.

### 2.10. Different Binding Affinity Between CIGB-258 and E18-3 to HDL

**Figure 10:**
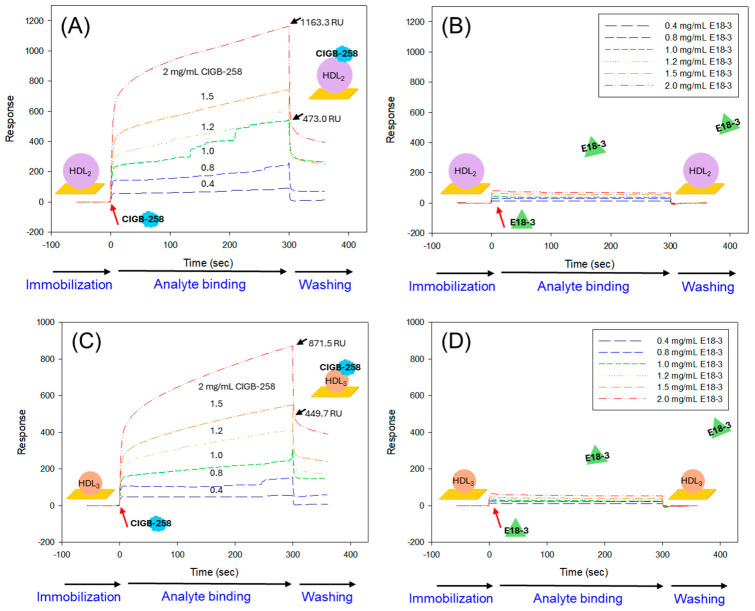

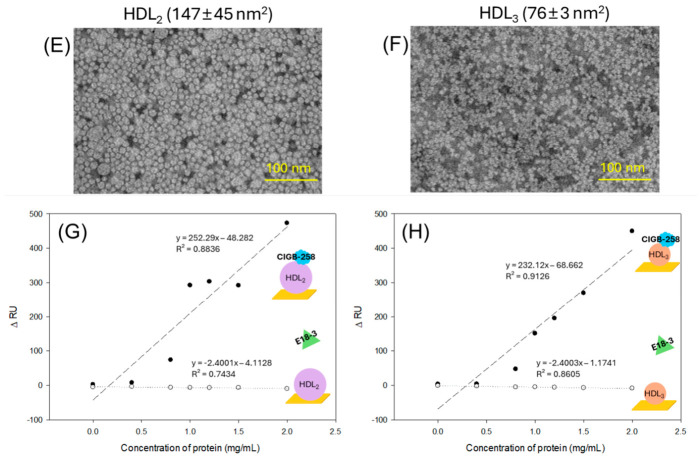
A comparative binding affinity of CIGB-258 and E18-3 with high-density lipoproteins (HDL_2_ and HDL_3_) determined by the surface plasmon resonance approach. (**A**,**B**) Binding interaction kinetics of CIGB-258 and E18-3 at concentrations of 0–2 mg/mL, respectively, with immobilized HDL_2_. (**C**,**D**) Binding interaction kinetics of CIGB-258 and E18-3 at different concentrations (0–2 mg/mL), respectively, with immobilized HDL_3_. (**E**,**F**) Transmission electron microscope (TEM) images of HDL_2_ and HDL_3_, respectively (scale bar, 100 nm); 150,000× magnified images. (**G**,**H**) ΔRU (difference in response units (RU) at the end of the analyte-binding phase and the reference RU) for CIGB-258 and E18-3 at tested concentrations (0–2 mg/mL) with HDL_2_ and HDL_3,_ respectively.

As depicted in Figure 10A, CIGB-258 exhibited a highly dynamic and potent binding interaction with the HDL_2_ surface. At the utmost used concentration (2.0 mg/mL), the maximal experimental response reached 1163.3 response units (RU) and 473.0 RU for attachment and association, respectively, whereas the dissociation experimental response was 690.3 RU, and the calculated theoretical maximum response (R_max_) was 145.8 ± 2.5 RU. In addition, a 14.8 µM^−1^s^−1^ association rate constant (K_a_), 0.35 s^−1^ dissociation rate constant (K_d_), and 0.024 µM equilibrium dissociation constant (*K_D_*) was quantified for CIGB-258 and HDL_2_ complex (Table 1). On the other hand, E18-3 showed no interaction with HDL_2_ at any of the tested concentrations (Figure 10B, Table 1).

Compared to HDL_2_, CIGB-258 showed a relatively lower kinetic interaction with HDL_3_ as reflected by a substantially reduced maximal experimental response of 871.5 RU and 449.7 RU for attachment and association, and 421.8 RU for the dissociated experimental response (Figure 10C). Consistently lower K_a_ and K_d_ values were noticed for the interaction of CIGB-258 with HDL_3_ compared to its interaction with HDL_2_ (Table 1). A higher *K_D_* (0.035 µM) was calculated for CIGB-258 to HDL_3_ than to HDL_2_ (0.024 µM), highlighting a 1.5-fold weaker affinity of CIGB-258 for HDL_3_ relative to HDL_2_ (Table 1). Contrary to this, no substantial interaction between HDL_3_ and E18-3 was noticed (Figure 10D, Table 1).

The TEM analysis revealed distinct morphologies and sizes for HDL_2_ and HDL_3_, which were used for the SPR analysis. Compared to the HDL_3,_ HDL_2_ showed a 1.96-fold larger average particle size_,_ reflecting a higher interaction area per particle (Figure 10E,F). Furthermore, CIGB-258 showed higher ΔRU (the difference between the RU at the end of the analyte-binding phase and the reference RU), for HDL_2_ than for HDL_3_ at each CIGB-258 concentration (0.4–2.0 mg/mL), underscoring that HDL_2_ has more surface area for binding than HDL_3_ (Figure 10G,H). In contrast, E18-3 did not show detectable ΔRU for HDL_2_ or HDL_3_ at any phase, from analyte binding to the washing phase, or at any tested concentration (0.4–2.0 mg/mL), highlighting the lack of binding interaction of E18-3 with HDL_2_ and HDL_3_.

## 3. Discussion

Intense inflammation is associated with severe organ damage and the induction of sepsis-like conditions [25]. The intense involvement of various cytokines and neutrophil efflux liberates a variety of proteases and ROS, aggravating the impact on sepsis [26]. Advanced end glycation products (AGEs), such as CML, generate ROS, induce oxidative stress, and activate NFκB, thereby inducing inflammation [27]. CML is known as an external stressor that can induce cytokine storm in kidney disease and type II diabetes [28]. Although endotoxins like LPS can cause sepsis, exposure to AGEs and ethanol substantially exacerbates sepsis by altering the activation of dendritic cells, monocytes, and macrophages [27,29]. In particular, ethanol’s impact on innate immunity exacerbates morbidity and mortality of sepsis [30]. In the present study, we observed a significant adverse effect of CML on the survival and swimming activity of zebrafish, which was further aggravated in the presence of Et-OH (i.e., CML+Et-OH), highlighting the synergistic effect of this combination. The reduced survivability and delayed recovery of swimming behavior may primarily be attributed to severe inflammation and oxidative stress induced by combined exposure. However, the exact molecular mechanisms underlying these effects remain unclear and warrant further investigation. The co-exposure of CIGB-258 substantially protected the acute death caused by CML+Et-OH; however, no substantial effect of the wild peptide E18-3 was noticed on CML+Et-OH-triggered mortality. The anti-inflammatory effects of CIGB-258 are the main reason for preventing CML+Et-OH-induced death and impaired swimming ability in zebrafish, as an inverse association between inflammation and mortality has been established [15,31]. Furthermore, the positive impact of TNF-α and IL-6 inhibitors infliximab and tocilizumab on the survival and swimming ability of hyperinflammatory zebrafish has been described [15]. The established anti-inflammatory role of CIGB-258 has been recognized, which inhibits various cytokines, including TNF-α, and activates T_reg_ cells [9,11], while the wild E18-3 failed to do so [11], thereby strengthening the present outcomes of higher survivability in the CIGB-258 groups. Despite this, a detailed mechanistic study is needed to establish the precise effect of CIGB-258 on CML+Et-OH adverse events.

CML has been shown to induce oxidative stress and to negatively affect antioxidants [32]. Consistently, the heightened MDA and diminished plasma sulfhydryl levels, an important indicator of oxidative damage [33], and diminished antioxidant activities (FRA and PON) were observed in the CML+Et-OH-treated group, underscoring the oxidative stress environment. The effect of CIGB-258 on oxidative stress is not well explored; however, a few studies suggest that CIGB-258 may prevent oxidative damage to lipoproteins from external stress [31]. In addition, CIGB-258 displayed a substantial protective effect, preventing zebrafish from apoptotic death by inhibiting CML-induced ROS [15], thereby strengthening the present findings of CIGB-258’s positive effect on CML+Et-OH-induced oxidative stress.

PON is an important HDL-associated antioxidant [34], and its activity is highly influenced by the structural variation of HDL [35]. CML is known to cause glycation of HDL, which, in turn, substantially alters its structure and function [36]. The effect of CIGB-258 has been documented to protect HDL from glycation [31], thereby preserving PON activity. The antiglycation effect of CIGB-258 might be an important reason for the higher PON activity in the CIGB-258-treated group. In contrast to CIGB-258, there are no reports on the effects of E18-3 on oxidative stress or HDL stability. The anti-inflammatory activity of CIGB-258 [9,12] is one reason for the lower oxidative stress in the CIGB-258 group, as a direct association between inflammation and oxidative stress has been established [37,38]. In line with this notion, the high oxidative stress in the E18-3 group can be explained by its inability to substantially modulate inflammation [11].

CML has been recognized to cause oxidative stress and inflammation, and a direct association between inflammation and the development of metabolic disease and dyslipidemia has been mentioned [39,40]. In addition, oxidative stress and central metabolism pathways have been shown to influence epigenetic modulation in the context of inflammation [41]. We also observed severe dyslipidemia (elevations in TC, TG, and LDL-C, and a decrease in HDL-C) in response to CML+Et-OH, which was substantially prevented by CIGB-258 treatment; however, no effect of E18-3 on dyslipidemia was observed. The substantial anti-inflammatory effect of CIGB-258 is likely a key contributor to counteracting CML+Et-OH-induced dyslipidemia. This statement is supported by earlier reports that identified a direct relationship between pro-inflammatory IL-6 and elevated TG levels [42,43]. Also, inflammation negatively affects HDL-C levels and HDL functionality. Several studies underscore elevated TNF-α and IL-6 levels, reduced HDL-C levels [40,44,45], and reduced HDL functionality, as evidenced by inferior PON activity [40]. The results, aligned with previous reports, suggest that CIGB-258 maintains TG levels and HDL functionality compromised by stress.

CML has been reported to cause hepatic steatosis, and a RAGE-dependent inflammatory response leads to hepatic damage [46]. Likewise, fatty liver changes, neutrophil infiltration, and elevated IL-6 levels indicated hepatic damage and inflammation in the CML+Et-OH-treated group. The CML+Et-OH-induced adverse effect in the liver was substantially altered by the treatment of CIGB-258, which is in accordance with the previous reports describing the effect of CIGB-258 on hepatic health and inflammation [15]. Contrary to the standard IL-6 inhibitor (tocilizumab) and TNF-α inhibitor (infliximab), CIGB-258 showed a greater effect in protecting against liver inflammation and damage [15]. In addition to the notable effect of hepatic inflammation, diminished ROS levels in the CIGB-258-treated group attest to improved hepatic health, as oxidative stress has been linked to a variety of liver disorders, including fatty liver [47]. Also, the reduced ROS levels observed following CIGB-258 treatment may contribute to the decreased IL-6 production in the liver, as ROS are widely recognized as a major mediator of inflammatory responses [48]. Better liver health can be linked to CIGB-258’s impact on HDL functionality and stability, as the immune-modulatory effect of HDL has been recognized [49] as regulating inflammation and has a positive impact on sepsis [49,50]. Contrary to CIGB-258, no effect of E18-3 was observed on CML+Et-OH-induced liver damage. The inability of E18-3 to mitigate CML+Et-OH-induced inflammation and ROS generation in the liver is probably the reason for its non-response to CML+Et-OH-induced hepatic damage.

Like its positive effect on the liver, CIGB-258 also showed substantial effects on CML+Et-OH-induced kidney damage, ROS production, and senescence. The notable suppression of kidney ROS generation by CIGB-258 appears to play a crucial role in preventing kidney damage caused by CML+Et-OH exposure. This statement is consistent with earlier reports suggesting a strong association between ROS and kidney health [51]. In addition, lower senescence in the kidney was associated with lower ROS levels in the CIGB-258-treated group. This notion aligns with previous reports suggesting that ROS and oxidative stress are key instigators of cellular senescence [52,53]. In contrast to CIGB-258, E18-3 had no effect on ROS or senescence and thus showed no kidney-protective effect against the acute toxicity caused by CML+Et-OH.

The intestinal histology revealed severe intestinal damage and bleeding in the CML+Et-OH group. The treatment of CIGB-258 effectively mitigated these changes, whereas E18-3 showed no substantial effect. The published report revealed an adverse effect of CML on tissue fibrosis [54]; likewise, a high level of tissue fibrosis was observed in the intestine of the CML+Et-OH group. Among the various events, the strong, provocative effect of CML on oxidative stress and inflammation can be linked to intestinal fibrosis. This notion is consistent with reports suggesting a clear effect of oxidative stress and inflammation on fibrosis [55,56]. A lower level of ROS in the CIGB-258-treated group, contrary to the higher ROS in the fibrotic area of the CML+Et-OH group, confirms the key involvement of oxidative stress with fibrosis and intestinal damage. In contrast to CIGB-258, E18-3 failed to inhibit ROS generation and consequently failed to provide any protective effect against CML+Et-OH-driven intestinal damage and fibrosis.

HDL is an important molecule associated with a variety of beneficial functions, including antioxidants and anti-inflammatory action. An adverse effect of CML on the glycation, structural stability, and functionality of HDL has been recognized [15,31]. Fortunately, the impact of CIGB-258 on the structural stability and functionality of HDL has been recognized [15,31]. However, the binding interactions, specifically the effect of CIGB-258 on HDL subpopulations (HDL_2_ and HDL_3_), have not been documented. In the present study, CIGB-258 showed high binding affinity for both HDL_2_ and HDL_3_, thereby maintaining their structural stability and functionality. In contrast, E18-3 failed to show a substantial affinity for HDL. The positive interaction of CIGB-258 with HDL helps to stabilize HDL functionality and therefore prevents CML+Et-OH-induced inflammation and oxidative damage. This statement is endorsed by numerous reports that identified distinct positive effects of HDL on inflammation and oxidative stress [57,58,59].

Limitation of this study: The study clearly establishes the functional superiority of CIGB-258 over E18-3 in mitigating CML+Et-OH-triggered events. However, the lack of plasma inflammatory marker analysis emerges as a basic limitation of the study. Furthermore, the effects of CIGB-258 and E18-3 on inflammation-related genes and proteins at the molecular level were not investigated, which remains another limitation of the present study. Future studies will address this limitation to better understand molecular events mediated by CIGB-258.

## 4. Materials and Methods

### 4.1. Materials

E18-3 and CIGB-258^®^ (Jusvinza^®^), a peptide of 27 amino acids derived from heat shock protein (HSP) 60, was provided for the research by the Center of Genetic Engineering and Biotechnology (CIGB), Havana, Cuba. Both peptides (E18-3 and CIGB-258^®^) had ~98% purity, assessed by reverse-phase high-performance liquid chromatography (RP-HPLC), and identity was confirmed by electrospray ionization mass spectrometry (ESI-MS). The isoelectric points of CIGB-228 and E18-3 were 6.83 and 4.58, respectively. A certificate of analysis (documenting purity) is provided in Supplementary Figures S1 and S2. *N*-ε-carboxylmethyllysine (CAS-No. 5746-04-3, Cat#14580-5g, Supplementary Figure S3), dihydroethidium (CAS-No. 104821-25-2, Cat #37291), acridine orange hydrochloride (CAS-No. 65-61-2, Cat#A9231), and oil red O (CAS-No. 1320-06-5, Cat#O0625) were procured from Sigma-Aldrich (St. Louis, MO, USA). All the other chemicals and reagents were of analytical grade and used as supplied unless otherwise stated.

### 4.2. Rearing of Zebrafish and Induction of Acute Inflammation

Fourteen-week-old zebrafish (wild-type AB strain) were reared in a water tank equipped with a circulating water system at 28 °C under a 14 h light and 10 h dark photoperiod and fed a normal diet (Tetra GmbH, Melle, Germany) twice a day (9 a.m. and 6 p.m.). The zebrafish were reared by adopting the standard guidelines of the Committee of Animal Care and Use of Raydel Research Institute (approval code: RRI-24-001, date of approval 2 September 2024). The laboratory water used for the rearing of zebrafish had a pH of 7.3, turbidity of 16 NTU, 0.18 mg/mL chlorine, 8 mg/mL DO, total bacterial counts ≤ 100 CFU and no presence of coliforms. Water quality analysis was conducted by Kirim Life Science Co., Ltd. (Daegu, Republic of Korea), which certified the water quality as safe for human and animal use (Supplementary Figure S4). After 1 week of acclimatization in the above-mentioned conditions, zebrafish (*n* = 180) were randomly distributed into six tanks (*n* = 30/group). Further, zebrafish from each group (*n* = 30) were randomly allocated into three tanks (*n* = 10/tank). Zebrafish [*n* = 30 (10 × 3 tanks)] of group I (control) and II were intraperitoneally injected with 10 μL of phosphate-buffered saline (PBS) and 50% ethanol (Et-OH) in PBS, respectively. Zebrafish in groups III and IV were intraperitonially injected with 10 μL of carboxymethyllysine (CML, 3 mM) in PBS and CML dissolved with 50% Et-OH (CML+Et-OH), respectively. Group V and VI zebrafish were intraperitoneally injected with 10 μL of CML+Et-OH in the presence of peptide E18-3 (1 μM) and CIGB-258 (1 μM), respectively. The used doses of CIGB-258 and CML were selected based on our previous reports [31,60]. Notably, no adverse effect of exposure to CIGB-258 alone was observed on zebrafish survivability or blood biochemical markers as reported previously [60].

The body weight of zebrafish across all groups was examined prior to treatment and 30, 60, and 180 min post-treatment using an electronic weighing machine (Ohaus, Parsippany-Troy Hills, NJ, USA).

### 4.3. Survivability and Swimming Analysis

Across all groups, the survival and swimming behavior of zebrafish were analyzed at 5, 30, 60 and 90 min post-intraperitoneal injection. Tail fin movement and paucity of body paroxysm were analyzed to assess swimming ability [61]. Gill movements, stagnant position, head upside or downside, and floating on the surface or sinking to the bottom of the tank were analyzed as markers of death following the guidelines of OECD 2019 [62].

### 4.4. Blood and Organ Collection and Quantification of Intestinal Bleeding

One hundred eighty minutes after treatment, blood and organs from zebrafish across all groups were collected. Zebrafish were sacrificed using hypothermic shock, and immediately, blood was collected. Blood was collected separately from zebrafish maintained in the three different tanks (n = 3) for each experimental group (as described in Section 4.2). For each tank within a given group, the collected blood was pooled into a single tube and mixed with ethylenediaminetetraacetic acid (EDTA, 1 mM) at a 2:3 (*v*/*v*) ratio. Blood samples were centrifuged at 6000 rpm (for 10 min), and the plasma was collected and stored in a refrigerator for further analysis.

Organs like the liver, kidney, and intestine were surgically recovered under microscope (Motic SMZ 168; Hong Kong, China) and stored in 10% formalin solution.

Images of the surgically incised intestines were captured using a microscope (Motic SMZ 168; Hong Kong, China). The captured images were processed for bleeding area quantification using the SPSS platform (https://image.net/ij, version 1.53, accessed on 17 June 2025) with a red color threshold of 20–120 to improve visibility of the bleeding area.

### 4.5. Plasma Biochemical Analysis

Plasma from the different groups was analyzed for malondialdehyde (MDA) levels, sulfhydryl content, ferric ion reduction ability (FRA), and paraoxonase (PON) activity using the previously described method [63]. A detailed methodology is provided in Supplementary Method Section S1. Blood glucose was quantified using an automated blood glucose meter (AccuCheck, Roche, Basel, Switzerland).

The commercial kits procured from Asan Pharmaceutical, Hwasung, Republic of Korea, were used to quantify plasma levels of total cholesterol (TC, AM 202-K), triglycerides (TG, AM 157-K), high-density lipoprotein cholesterol (HDL-C, AM 203-K), aspartate aminotransferase (AST, AM 102K), and alanine aminotransferase (ALT, AM 103-K), following the manufacturers’ guidelines. A detailed methodology is provided in Supplementary Method Section S2.

### 4.6. Hematoxylin and Eosin (H&E), Oil Red O (ORO) and Immunohistology (IHC) Staining

Tissue (liver, kidney, and intestine) was blocked with FSC22 frozen solution (Leica, Nussloch, Germany) and processed for sectioning (7 μm thick) using a cryo-microtome (Leica CM-1510S, Nussloch, Germany). The morphological changes in the tissue section were assessed by H&E staining [64]. In brief, the tissue section was incubated with Ventana HE 600 hematoxylin (lot no. N11615; Roche, Tucson, AZ, USA) for 5 min, rinsed with water for 1 min, and then treated with 0.5% HCl. After ~20 s of water wash, the section was exposed to 0.05% ammonia water for ~10 s, followed by a through-water rinse. The section was then stained with Ventana HE 600 eosin (lot no. H304444; Roche, Tucson, AZ, USA) for 1 min. Finally, the section was washed with ethanol, air-dried, and visualized under a microscope.

For the evaluation of fatty liver, the liver section (7 μm) was covered with ORO solution (0.5 mL) and stained for 5 min at 60 °C. After incubation, the excess stain was removed by rinsing the section using 60% isopropanol. The section was air-dried and examined under a microscope.

Interleukin (IL)-6 in the liver was detected by IHC staining. The liver section (7 μm) was flooded with 200× diluted anti-IL6 antibody (mouse IgG ab9324 Abcam, Cambridge, UK), and the section was incubated for 16 h at 4 °C. Subsequently, the section was washed with PBS (2 times) and processed for development using the EnVision HRP-polymer kit (Dako, Glostrup, Denmark), which contains a horseradish peroxidase (enzyme)-linked secondary antibody (anti-mouse IgG, Dako, Glostrup, Denmark, K4003, lot no. 11455874) and a chromogenic substrate (developmental solution K3468, lot no. 11775820, Dako, Glostrup, Denmark).

### 4.7. Senescence, Dihydroethidium (DHE), and Masson-Trichome Staining

Cellular senescence and ROS generation were detected by senescence-associated β-galactosidase (SA-β-gal) staining and DHE fluorescence staining. For SA-β-gal staining, 0.75 mL of 0.1% 5-bromo-4-choloro-3-indolyl-β-galactopyranoside solution (X-gal) was applied to the tissue section. After 16 h of incubation in a moist atmosphere, the section was washed with water. The section was air-dried and visualized under a microscope to detect blue-stained SA-β-gal-positive cells.

For the DHE staining, 0.25 mL of 30 μM DHE solution was applied to the tissue section. After 5 min of incubation at RT, the section was washed and analyzed under a fluorescent microscope (Nikon Eclipse TE2000, Tokyo, Japan).

Intestinal tissue fibrosis was detected by Masson-trichrome staining, following the method described earlier, with slight modifications [65]. The intestinal section (7 μm thick) was immersed in Weigert’s iron hematoxylin solution [prepared by mixing equal proportions of solution A (4 g hematoxylin in 200 mL of 80% ethanol) and solution B (8 g FeCl_3_ in 190 mL distilled H_2_O and 2 mL of HCl)]. After 5 min staining in the dark, the section was washed three times with distilled H_2_O and subsequently immersed in Biebrich scarlet acid fuchsin solution (prepared by dissolving 2.25 g Biebrich scarlet and 0.25 g acid fuchsin in 250 mL distilled H_2_O containing 2 mL glacial acetic acid). After 5 min, the section was washed 3 times with distilled H_2_O and subsequently immersed in 1% phosphomolybdic acid. Following 2 min incubation, the section was treated for 5 min in 1.8% of aniline blue solution (prepared by dissolving 4.5 g of aniline blue in 250 mL distilled H_2_O containing 4.5 mL of glacial acetic acid). The section was rinsed with distilled H_2_O, followed by 30 s exposures to a 1% acetic acid solution. Finally, the section was rinsed twice with distilled H_2_O and visualized under a microscope (Nikon, Tokyo, Japan).

### 4.8. Isolation of Human High-Density Lipoprotein (HDL)

Blood was collected from three healthy male volunteers (aged 25 ± 3 years) after a 12 h fasting period. Informed consent was obtained from all subjects involved in the study. The study was conducted in accordance with the Helsinki guidelines and approved by the Institutional Board of the Korea National Institute for Bioethics Policy (KoNIBP, approval number P01-202109-31-009, approval date 27 September 2021). All participants provided voluntary informed consent. The HDL from the blood was isolated using sequential density gradient ultracentrifugation [66]. In brief, 5 mL of the serum was adjusted to the density (1.019 < d < 1.063 g/mL) and ultracentrifuged at 100,000× *g*. After 24 h of centrifugation, the VLDL and LDL fractions were removed, and the remaining serum density was adjusted to 1.063 < d < 1.125 g/mL. After 24 h centrifugation at 100,000× *g,* the HDL_2_ fraction was recovered. The density of the remaining serum was adjusted to 1.125 < d < 1.225 g/mL, followed by 100,000× *g* centrifugation for 24 h to recover the HDL_3_ fraction. The density was adjusted using NaCl and NaBr using the standard method [67]. The isolated HDL_2_ and HDL_3_ were dialyzed against Tris-buffered saline (TBS, pH 8.0) to remove salt. After 24 h of dialysis, the HDL_2_ and HDL_3_ were stored in the refrigerator for further analysis.

### 4.9. Immobilization of HDL on the SPR Sensor Chip

To determine the optimal immobilization conditions, pH scanning was performed using human HDL_2_ and HDL_3_ at two different concentrations (1 and 5 mg/mL). The HDL_2_ and HDL_3_ were dissolved in a degassed TBS buffer (10 mM Tris, 140 mM NaCl, and 1 mM EDTA, pH 8.0). The solution was individually diluted [1:10 (*v/v*)] with 10 mM sodium acetate buffer at pH 4.5, 5.0, and 5.5 (Cytiva, Marlborough, MA, USA, Cat # BR100350, BR100351, and BR100352, respectively), and used for pH scouting. Based on the pre-concentration profile monitoring [in a surface plasmon resonance (SPR) sensor], pH 4.5 was selected as the optimal immobilization buffer to provide a significant response.

HDL_2_ (approximately MW = 360,000) or HDL_3_ (approximately MW = 175,000), each with 0.1 mg/mL protein, was covalently immobilized onto a surface plasmon resonance (SPR) sensor chip using a standard amine-coupling chemistry kit (Cytiva, Marlborough, MA, USA, Cat # BR100050). Before the immobilization reaction, the sensor surface was activated for 400 s with a 1:1 mixture of 0.4 M 1-ethyl-3-(3-dimethylaminopropyl) carbodiimide hydrochloride (EDC) and 0.1 M N-hydroxysuccinimide (NHS). Following activation, each HDL was injected over the surface for 300 s at a flow rate of 5 µL/min at room temperature (RT). This coupling reaction resulted in a significant increase in the response signal, reaching approximately 35,477 ± 462 RU and 29,432 ± 328 RU, for HDL_2_ and HDL_3_, respectively. To remove any non-covalently bound or physically adsorbed HDL, the chip was thoroughly washed with degassed TBS buffer for 300 s. Such a high ligand density was targeted to ensure a sufficient signal-to-noise ratio during binding of relatively small analytes (CIGB-258 and E18-3) and to maintain a robust, stable baseline throughout multiple regeneration cycles.

### 4.10. Different Binding Affinity Between CIGB-258 and E18-3 to HDL

To compare the binding affinity of CIGB-258 and E18-3 to HDL, SPR experiments were carried out using a Biacore T200 SPR instrument (Cytiva, Uppsala, Sweden) equipped with a Series S Sensor Chip CM5 (Cytiva, Uppsala, Sweden). Data acquisition and analysis were carried out using the Biacore T200 control software (version 2.0.2) and the Biacore T200 evaluation software (version 3.1), respectively. All experiments were conducted at the National NanoFab Center (NNFC), located at the Korea Advanced Institute of Science and Technology (KAIST, Daejeon, Republic of Korea).

For kinetic analysis, CIGB-258 and E18-3 were prepared in a series of concentrations (0, 0.4, 0.8, 1.0, 1.2, 1.5, and 2.0 mg/mL) in the TBS buffer. Each analyte was injected at a flow rate of 5 µL/min over the immobilized HDL surface at RT using a single-cycle kinetic mode. The analytes were delivered for up to 300 s to monitor the association and dissociation phases. The produced sensorgrams based on analyte (CIGB-258 and E18-3) concentration were analyzed to determine the binding affinity (*K_D_*), association rate constants (Ka), and dissociation rate constants (Kd). After completion of the experiment, the sensor was regenerated for subsequent use by detaching the bound analyte using 3M urea.

### 4.11. Transmission Electron Microscopy (TEM)

Five μL of HDL_2_/HDL_3_ samples (0.2 mg/mL in TBS) were mixed with 5 μL of 2% sodium phosphotungstate (pH 7.4) for negative staining [31]. Subsequently, 5 μL of the HDL_2_/HDL_3_ suspension was placed on a carbon-coated 300-mesh copper grid, air-dried for 2 min and incubated at 50 °C for 2 h. Finally, the sample was visualized under TEM (HT-7800, Hitachi, Tokyo, Japan) at 100 kV accelerating voltage, and the images were captured at 150,000× magnification.

### 4.12. Statistical Analysis

One-way analysis of variance (ANOVA) followed by Tukey’s post hoc analysis was used to compare multivariate data using the SPSS platform (version 29, Chicago, IL, USA). Normality of the data was assessed prior to performing the one-way ANOVA. For the comparison of the bivariate data a two-tailed *t*-test was utilized.

## 5. Conclusions

CIGB-258 substantially inhibits oxidative stress, augments HDL-C levels, and improves PON and FRA activity, leading to the rescue of zebrafish from acute death and swimming impairment induced by CML+Et-OH. Whereas no effect of E18-3 was noticed to protect the zebrafish from CML+Et-OH-provoked acute toxicity, CIGB-258 inhibited hepatic inflammation, steatosis, ROS production, and renal senescence, and minimized gastrointestinal oxidative injury, bleeding, and fibrosis. Also, CIGB-258 showed substantial affinity for HDL, whereas no interaction of E18-3 was observed for HDL. The study concludes that CIGB-258 has a distinct positive effect compared to E18-3 to counter CML+Et-OH-induced sepsis-like events by modulating oxidative stress, inflammation, and HDL functionality to protect multiple organ damage and acute death in zebrafish.

## Figures and Tables

**Table 1:** Binding constants for CIGB-258 and E18-3 interactions with high-density lipoproteins (HDL_2_ and HDL_3_).

Binding Constants	HDL_2_ (MW = 360,000)		HDL_3_ (MW = 175,000)	
	CIGB-258	E18-3	CIGB-258	E18-3
K_a_ (µM^−1^s^−1^)	14.78	0.1	6.20	0.1
K_d_ (s^−1^)	0.35	0.003	0.22	0.002
*K_D_* (µM)	0.024	ND	0.035	ND
R_max_ ^1^	145.8 ± 2.5	0.0025	220.3 ± 4.0	0.0025

^1^ The sensorgrams were fitted to a 1:1 Langmuir binding model to determine the kinetic parameters with analysis software (Biacore T200 Evaluation Software, version 3.1, Cytiva, Uppsala, Sweden). ND, not detected. K_a,_ K_d_, and *K_D_* represent association rate constant, dissociation rate constant, and equilibrium dissociation constant, respectively.

## Data Availability

The data used to support the findings of this study are available from the corresponding author upon reasonable request.

## Supplementary Materials

The following supporting information can be downloaded at: https://www.mdpi.com/article/10.3390/ijms27104516/s1.

## Abbreviations

The following abbreviations are used in this manuscript:

AGE	Advanced glycation end products
ALT	Alanine aminotransferase
AO	Acridine orange
AST	Aspartate aminotransferase
CML	Carboxymethyllysine
DHE	Dihydroethidium
Et-OH	Ethanol
FRA	Ferric ion reduction activity
HDL-C	High-density lipoprotein cholesterol
HSP	Heat shock protein
IL-6	Interleukin 6
K_a_	Association rate constant
K_d_	Dissociation rate constant
*K_D_*	Equilibrium dissociation constant
LDL-C	Low-density lipoprotein cholesterol
MDA	Malondialdehyde
NFκB	Nuclear factor kappa-light-chain-enhancer of activated B cells
ORO	Oil red O
PBS	Phosphate-buffered saline
PON	Paraoxonase
RAGE	Receptors for advanced glycation end products
R_max_	Theoretical maximum response
ROS	Reactive oxygen species
RP-HPLC	Reverse-phase high-performance liquid chromatography
RU	Response unit
SA-β-gal	Senescent-associated β-galactosidase
SPR	Surface plasmon resonance
TBS	Tris-buffered saline
TC	Total cholesterol
TEM	Transmission electron microscopy
TG	Triglycerides
TLR-4	Toll-like receptor-4
TNF-α	Tumor necrosis factor alpha
T_reg_	Regulatory T cells

## References

[1] Shin K.S. (2025). Current and emerging biomarkers for sepsis: Diagnostic and prognostic tools. Biomed. Sci. Lett..

[2] Fajgenbaum D.C., June C.H. (2020). Cytokine storm. N. Engl. J. Med..

[3] Dasu M.R., Devaraj S., Park S., Jialal I. (2010). Increased toll-like receptor (TLR) activation and TLR ligands in recently diagnosed type 2 diabetic subjects. Diabetes Care.

[4] Buetler T.M., Leclerc E., Baumeyer A., Latado H., Newell J., Adolfsson O., Parisod V., Richoz J., Maurer S., Foata F. (2008). Nε-carboxymethyllysine-modified proteins are unable to bind to rage and activate an inflammatory response. Mol. Nutr. Food Res..

[5] Ahmad S., Khan H., Siddiqui Z., Khan M.Y., Rehman S., Shahab U., Godovikova T., Silnikov V., Moinuddin (2018). AGEs, RAGEs and s-RAGE; friend or foe for cancer. Semin. Cancer Biol..

[6] Zhou M., Zhang Y., Shi L., Li L., Zhang D., Gong Z., Wu Q. (2024). Activation and modulation of the AGEs-RAGE axis: Implications for inflammatory pathologies and therapeutic interventions—A Review. Pharmacol. Res..

[7] Wu X., Shi X., Chen X., Yin Z. (2023). Advanced glycation end products regulate the receptor of AGEs epigenetically. Front. Cell Dev. Biol..

[8] Zininga T., Ramatsui L., Shonhai A. (2018). Heat shock proteins as Immunomodulants. Molecules.

[9] Domínguez-Horta M.d.C., Serrano-Díaz A., Hernández-Cedeño M., Martínez-Donato G., Guillén-Nieto G. (2023). A peptide derived from hsp60 reduces proinflammatory cytokines and soluble mediators: A therapeutic approach to inflammation. Front. Immunol..

[10] Cabrales-Rico A., Ramos Y., Besada V., Del Carmen Domínguez M., Lorenzo N., García O., Alexis J., Prada D., Reyes Y., López A.M. (2017). Development and validation of a bioanalytical method based on LC-MS/MS analysis for the quantitation of CIGB-814 peptide in plasma from Rheumatoid Arthritis patients. J. Pharm. Biomed. Anal..

[11] Dominguez M.d.C., Lorenzo N., Barbera A., Darrasse-Jeze G., Hernández M.V., Torres A., Hernández I., Gil R., Klatzmann D., Padrón G. (2011). An altered peptide ligand corresponding to a novel epitope from heat-shock protein 60 induces regulatory T cells and suppresses pathogenic response in an animal model of adjuvant-induced arthritis. Autoimmunity.

[12] Hernández-Cedeño M., Venegas-Rodriguez R., Peña-Ruiz R., Bequet-Romero M., Santana-Sanchez R., Penton-Arias E., Martinez-Donato G., Guillén-Nieto G., Dominguez-Horta M.d.C. (2021). CIGB-258, a peptide derived from human heat-shock protein 60, decreases hyperinflammation in COVID-19 patients. Cell Stress Chaperones.

[13] Cho K.-H., Kim J.-E., Kang D.-J., Dominguez-Horta M.d.C., Martinez-Donato G. (2024). Synergistic anti-inflammatory activity of apolipoprotein A-I and CIGB-258 in reconstituted high-density lipoproteins (rHDL) against acute toxicity of carboxymethyllysine in zebrafish and Its embryo. Pharmaceuticals.

[14] Cho K.-H., Bahuguna A., Lee Y., Lee S.H., Dominguez-Horta M.d.C., Martinez-Donato G. (2024). Synergistic anti-inflammatory activity of lipid-free apolipoprotein (apo) A-I and CIGB-258 in acute-phase zebrafish via stabilization of the apoA-I structure to enhance anti-glycation and antioxidant activities. Int. J. Mol. Sci..

[15] Cho K.-H., Nam H.-S., Kim J.-E., Na H.-J., del Carmen Dominguez-Horta M., Martinez-Donato G. (2023). CIGB-258 exerts potent anti-inflammatory activity against carboxymethyllysine-induced acute inflammation in hyperlipidemic zebrafish via the protection of apolipoprotein AI. Int. J. Mol. Sci..

[16] Cho K.-H., Lee Y., Lee S.H., Kim J.-E., Bahuguna A., Dominguez-Horta M.d.C., Martinez-Donato G. (2024). Enhancing wound healing and anti-Inflammatory effects by combination of CIGB-258 and apolipoprotein A-I against carboxymethyllysine toxicity in zebrafish: Insights into structural stabilization and antioxidant properties. Antioxidants.

[17] Mastrogiovanni M., Martínez-Navarro F.J., Bowman T.V., Cayuela M.L. (2024). Inflammation in development and aging: Insights from the zebrafish model. Int. J. Mol. Sci..

[18] Zanandrea R., Bonan C.D., Campos M.M. (2020). Zebrafish as a model for inflammation and drug discovery. Drug Discov. Today.

[19] Howe K., Clark M.D., Torroja C.F., Torrance J., Berthelot C., Muffato M., Collins J.E., Humphray S., McLaren K., Matthews L. (2013). The zebrafish reference genome sequence and its relationship to the human genome. Nature.

[20] Tan X., Liu H., Liang F., Chen G. (2026). Zebrafish as a multimodal platforms for anti-inflammatory phytomedicine discovery and translation. Front. Pharmacol..

[21] Szumlak A., Luchowska-Kocot D., Qtaishat F.A., Boguszewska-Czubara A. (2024). Creating a reliable zebrafish model for studying inflammation: Exploring the therapeutic potential of xanthohumol. Sci. Radices.

[22] He J., Xu P., Chen R., Chen M., Wang B., Xie Y., Yang Q., Sun D., Ji M. (2024). Exploiting the zebrafish model for sepsis research: Insights into pathophysiology and therapeutic potentials. Drug Des. Dev. Ther..

[23] Liu C., Li J., Wang D., Liu J., Liu K., Li P., Zhang Y. (2024). Recent advances of the zebrafish model in the discovery of marine bioactive molecules. Mar. Drugs.

[24] Widder M., Carbaugh C., van der Schalie W., Miller R., Brennan L., Moore A., Campbell R., Akers K., Ressner R., Martin M. (2024). Identification of potential sepsis therapeutic drugs using a zebrafish rapid screening approach. Life.

[25] Antar S.A., Mahmoud A.M., Abdo W., Gad C., Al-Karmalawy A.A. (2023). A comprehensive overview of organ inflammatory responses: Genesis, possible mechanisms, and mediators of inflammation. Pharm. Sci..

[26] Wiersinga W.J., van der Poll T. (2022). Immunopathophysiology of human sepsis. eBioMedicine.

[27] Baumann M. (2009). Advanced glycation endproducts in sepsis and mechanical ventilation: Extra or leading man?. Crit. Care.

[28] Tsekovska R., Gatev E., Mironova R., Kerezieva S., Ilieva S., Ilieva T., Vasileva B., Niwa T., Popova D., Vasilev V. (2025). Serum levels of *N*^ε^-(carboxymethyl)-lysine in chronic kidney disease and type 2 diabetes mellitus. Biomedicines.

[29] Roychowdhury S., Pant B., Cross E., Scheraga R., Vachharajani V. (2024). Effect of ethanol exposure on innate immune response in sepsis. J. Leukoc. Biol..

[30] O’brien J.M., Lu B., Ali N.A., Martin G.S., Aberegg S.K., Marsh C.B., Lemeshow S., Douglas I.S. (2007). Alcohol dependence is independently associated with sepsis, septic shock, and hospital mortality among adult intensive care unit patients. Crit. Care Med..

[31] Cho K.-H., Kim J.-E., Nam H.-S., Kang D.-J., Na H.-J. (2022). Anti-inflammatory activity of CIGB-258 against acute toxicity of carboxymethyllysine in paralyzed zebrafish via enhancement of high-density lipoproteins stability and functionality. Int. J. Mol. Sci..

[32] Boesten D.M.P.H.J., Elie A.G.I.M., Drittij-Reijnders M.-J., den Hartog G.J.M., Bast A. (2014). Effect of Nɛ-Carboxymethyllysine on oxidative Stress and the glutathione system in beta cells. Toxicol. Rep..

[33] van Zoeren-Grobben D., Lindeman J.H.N., Houdkamp E., Moison R.M.W., Wijnen J.T., Berger H.M. (1997). Markers of oxidative stress and antioxidant activity in plasma and erythrocytes in neonatal respiratory distress syndrome. Acta Paediatr..

[34] Kumar M., Ali W., Yadav K., Kaumri S., Mishra S., Nardi P., Iellamo F., Bernardini S., Pradhan A., Perrone M.A. (2024). High-density lipoprotein-associated paraoxonase-1 (PON-1) and scavenger receptor class B Type 1 (SRB-1) in coronary artery disease: Correlation with disease severity. J. Clin. Med..

[35] Ferretti G., Bacchetti T., Nègre-Salvayre A., Salvayre R., Dousset N., Curatola G. (2006). Structural modifications of HDL and functional consequences. Atherosclerosis.

[36] Kashyap S.R., Osme A., Ilchenko S., Golizeh M., Lee K., Wang S., Bena J., Previs S.F., Smith J.D., Kasumov T. (2018). Glycation reduces the stability of ApoAI and increases HDL dysfunction in diet-controlled Type 2 diabetes. J. Clin. Endocrinol. Metab..

[37] Ramos-González E.J., Bitzer-Quintero O.K., Ortiz G., Hernández-Cruz J.J., Ramírez-Jirano L.J. (2021). Relationship between inflammation and oxidative stress and its effect on multiple sclerosis. Neurología.

[38] Zuo L., Prather E.R., Stetskiv M., Garrison D.E., Meade J.R., Peace T.I., Zhou T. (2019). Inflammaging and oxidative stress in human diseases: From molecular mechanisms to novel treatments. Int. J. Mol. Sci..

[39] Abtin S., Ziveh T., Rezaee-Tavirani M. (2025). The complicated relationship between inflammation and metabolic dysfunction. J. Diabetes Metab. Disord..

[40] Esteve E., Ricart W., Fernández-Real J.M. (2005). Dyslipidemia and inflammation: An evolutionary conserved mechanism. Clin. Nutr..

[41] García-Giménez J.L., Cánovas-Cervera I., Nacher-Sendra E., Dolz-Andrés E., Sánchez-Bernabéu Á., Agúndez A.B., Hernández-Gil J., Mena-Mollá S., Pallardó F.V. (2025). Oxidative stress and central metabolism pathways impact epigenetic modulation in inflammation and immune response. Free Radic. Biol. Med..

[42] Qiao Q., Bouwman F.G., van Baak M.A., Roumans N.J.T., Vink R.G., Mariman E.C.M. (2022). Plasma levels of triglycerides and IL-6 are associated with weight regain and fat mass expansion. J. Clin. Endocrinol. Metab..

[43] Nonogaki K., Fuller G.M., Fuentes N.L., Moser A.H., Staprans I., Grunfeld C., Feingold K.R. (1995). Interleukin-6 stimulates hepatic triglyceride secretion in rats. Endocrinology.

[44] Mizia-Stec K., Zahorska-Markiewicz B., Mandecki T., Janowska J., Szulc A., Jastrzębska-Maj E., Gąsior Z. (2003). Hyperlipidaemias and serum cytokines in patients with coronary artery disease. Acta Cardiol..

[45] Skoog T., Dichtl W., Boquist S., Skoglund-Andersson C., Karpe F., Tang R., Bond M.G., de Faire U., Nilsson J., Eriksson P. (2002). Plasma tumor necrosis factor-α and early carotid atherosclerosis in healthy middle-aged men. Eur. Heart J..

[46] Gaens K.H.J., Niessen P.M.G., Rensen S.S., Buurman W.A., Greve J.W.M., Driessen A., Wolfs M.G.M., Hofker M.H., Bloemen J.G., Dejong C.H. (2012). Endogenous formation of Nε-(carboxymethyl) lysine is increased in fatty livers and induces inflammatory markers in an in vitro model of hepatic steatosis. J. Hepatol..

[47] Conde de la Rosa L., Goicoechea L., Torres S., Garcia-Ruiz C., Fernandez-Checa J.C. (2022). Role of Oxidative Stress in Liver Disorders. Livers.

[48] Mittal M., Siddiqui M.R., Tran K., Reddy S.P., Malik A.B. (2014). Reactive oxygen species in inflammation and tissue injury. Antioxid. Redox Signal..

[49] Trakaki A., Marsche G. (2021). Current understanding of the immunomodulatory activities of high-density lipoproteins. Biomedicines.

[50] Li M., Barros-Pinkelnig M., Arbous S.M., Christoffersen C., Rensen P., Kooijman S. (2025). High-density lipoprotein: A biomarker and therapeutic target in sepsis. Crit. Care.

[51] Irazabal M.V., Torres V.E. (2020). Reactive oxygen species and redox signaling in chronic kidney disease. Cells.

[52] Stojanovic B., Jovanovic I., Dimitrijevic Stojanovic M., Stojanovic B.S., Kovacevic V., Radosavljevic I., Jovanovic D., Miletic Kovacevic M., Zornic N., Arsic A.A. (2025). Oxidative stress-driven cellular senescence: Mechanistic crosstalk and therapeutic horizons. Antioxidants.

[53] Nousis L., Kanavaros P., Barbouti A. (2023). Oxidative stress-induced cellular senescence: Is labile iron the connecting kink?. Antioxidants.

[54] Lee B.H., Hsu W.H., Hsu Y.W., Pan T.M. (2013). Suppression of dimerumic acid on hepatic fibrosis caused from carboxymethyl-lysine (CML) by attenuating oxidative stress depends on Nrf2 activation in hepatic stellate cells (HSCs). Food Chem. Toxicol..

[55] Mack M. (2018). Inflammation and Fibrosis. Matrix Biol..

[56] Morry J., Ngamcherdtrakul W., Yantasee W. (2017). Oxidative stress in cancer and fibrosis: Opportunity for therapeutic intervention with antioxidant compounds, enzymes, and nanoparticles. Redox Biol..

[57] Tabet F., Rye K.A. (2009). High-density lipoproteins, inflammation and oxidative stress. Clin. Sci..

[58] Nazir S., Jankowski V., Bender G., Zewinger S., Rye K.-A., van der Vorst E.P. (2020). Interaction between high-density lipoproteins and inflammation: Function matters more than concentration!. Adv. Drug Deliv. Rev..

[59] Denimal D. (2024). Antioxidant and anti-inflammatory functions of high-density lipoprotein in type 1 and type 2 diabetes. Antioxidants.

[60] Cho K.-H., Lee Y., Lee S.H., Bahuguna A., Domínguez-Horta M.d.C., Martínez-Donato G. (2026). CIGB-258, a potential novel approach to treat sepsis-like hyperinflammation, reduces gastrointestinal Hemorrhage in zebrafish exposed to carboxymethyllysine and ethanol. Pharmaceuticals.

[61] Burris B., Jensen N., Mokalled M.H. (2021). Assessment of swim endurance and swim behavior in adult zebrafish. J. Vis. Exp..

[62] OECD (2019). Test No. 203: Fish, Acute Toxicity Testing. OECD Guidelines for the Testing of Chemicals.

[63] Cho K.-H., Kim J.-E., Lee M.-S., Bahuguna A. (2024). Oral supplementation of ozonated sunflower oil augments plasma antioxidant and anti-inflammatory abilities with enhancement of high-density lipoproteins functionality in rats. Antioxidants.

[64] Fischer A.H., Jacobson K.A., Rose J., Zeller R. (2006). Hematoxylin and eosin staining of tissue and cell sections. Basic Methods in Microscopy.

[65] Foot N.C. (1933). The Masson Trichrome Staining Methods in Routine Laboratory Use. Stain. Technol..

[66] Cho K.H., Nam H.S., Baek S.H., Kang D.J., Na H., Komatsu T., Uehara Y. (2023). Beneficial effect of Cuban policosanol on blood pressure and serum lipoproteins accompanied with lowered glycated hemoglobin and enhanced high-density lipoprotein functionalities in a randomized, placebo-controlled, and double-blinded trial with healthy Japanese. Int. J. Mol. Sci..

[67] Havel R.J., Eder H.A., Bragdon J.H. (1955). The distribution and chemical composition of ultracentrifugally separated lipoproteins in human serum. J. Clin. Investig..

