# Supplementary material for: Comparison of Anti-Acute Phase Effect of CIGB-258 and Its Wild-Type Peptide (E18-3) in a Hyperinflammatory and Acute Bleeding Model of Zebrafish: A Surface Plasmon Resonance Study to Compare Binding Affinity with High-Density Lipoproteins
Source: Int J Mol Sci. 2026 May 18;27(10):4516. doi: 10.3390/ijms27104516 (PMC13207133; doi:10.3390/ijms27104516)
Supplement: Supplementary file 1 [file ijms-27-04516-s001.zip › Supplementary Material_ ijms-4294580.pdf]

Supplementary Material

Supplementary Figure S1

CENTRO DE INGENIERÍA GENÉTICA Y BIOTECNOLOGÍA<br>SERVICIO DE PÉPTIDOS SINTÉTICOS<br>CARACTERIZACIÓN POR RP-HPLC DE PÉPTIDOS SINTETICOS<br>(CLIENTE)	065-027	PPO 5.65.005.14
	Edición 02	Hoja 1 de 1

Código del péptido: 023P141

Equipamiento: Shimadzu
Nº de Identificación: I-LFSHIMA1
Fecha de venc. calibración: 09/2024

Disoluciones:
(A) TFA/Agua (0,1%) Lote: A24002
(B) TFA/Acetonitrilo (0,05%) Lote: B24002

Gradiente Utilizado: 5% a 60% B en 35 min
Longitud de onda: 226 nm
Grado de pureza: 98.70%

Documentos adjuntos:
Cromatograma (s) de RP-HPLC ☑
Espectro (s) de masas ☑
MM: 2988,62 Da
Purificación: ✓
Fracciones puras mezcladas: ✓

Observaciones: Se entregaron 79.7 mg de péptido con 98.70% de pureza

Operario: Yazara C. Guerra Rojas
Cargo: Analista de Lab.
Supervisado por: David Diago Alvarez
Cargo: J' Lab Anal-Purif.
Recibido por: Anabel Serrano Díaz

Firma: [illegible]
Fecha: 16/01/2024
Firma: [illegible]
Fecha: 19/02/2024
Firma: [illegible]
Fecha: 19/02/2024

DOCUMENTO VIGENTE

Supplementary Figure S1: Certificate of E18-3 analysis.

Supplementary Figure S2

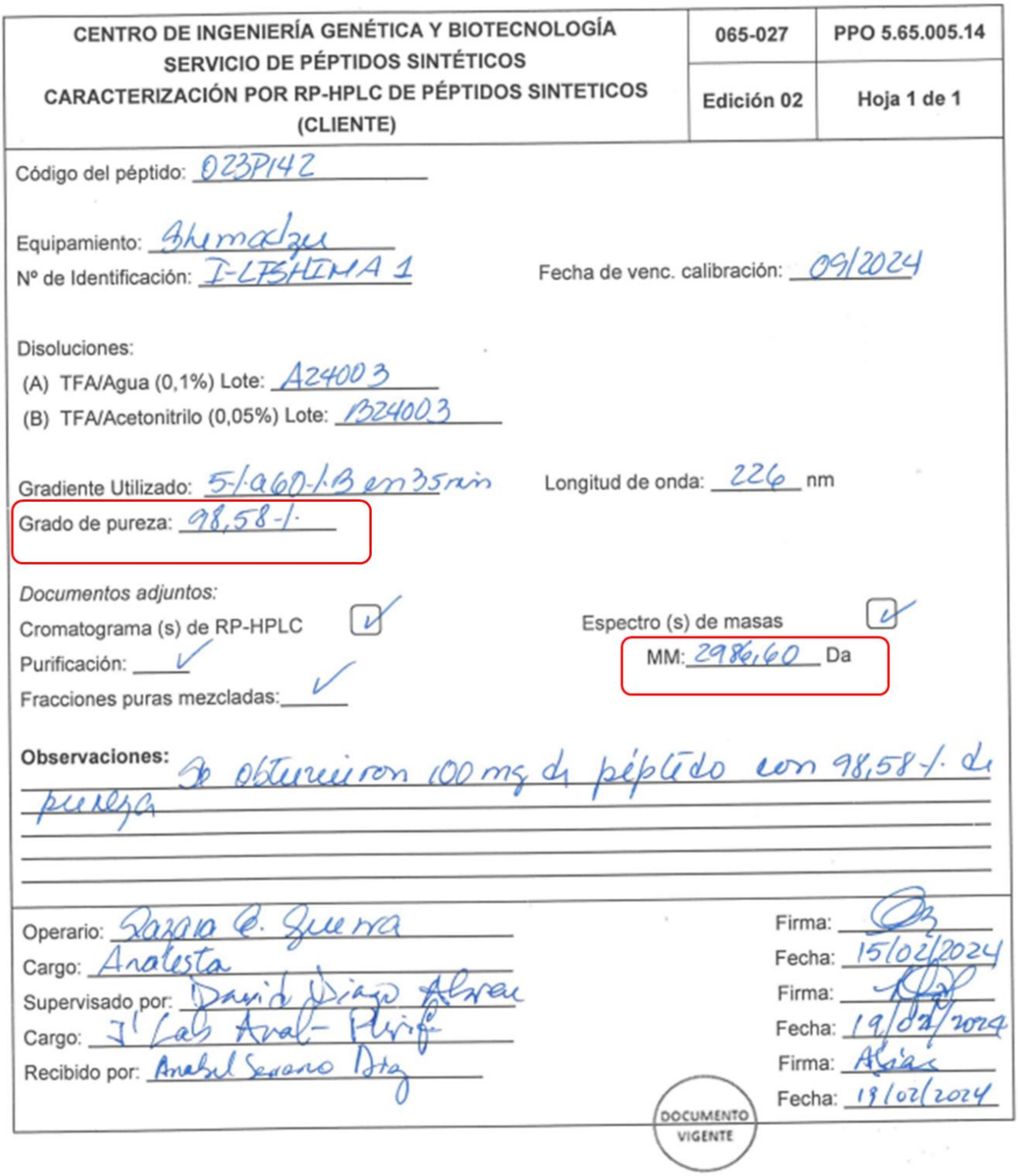

CENTRO DE INGENIERÍA GENÉTICA Y BIOTECNOLOGÍA<br>SERVICIO DE PÉPTIDOS SINTÉTICOS<br>CARACTERIZACIÓN POR RP-HPLC DE PÉPTIDOS SINTETICOS<br>(CLIENTE)	065-027	PPO 5.65.005.14
	Edición 02	Hoja 1 de 1

Código del péptido: 023P142

Equipamiento: Shimadzu

Nº de Identificación: I-LFSHIMA 1 Fecha de venc. calibración: 09/2024

Disoluciones:

(A) TFA/Agua (0,1%) Lote: A24003

(B) TFA/Acetonitrilo (0,05%) Lote: B24003

Gradiente Utilizado: 5% a 60% B en 35 min Longitud de onda: 226 nm

Grado de pureza: 98,58 %

Documentos adjuntos:

Cromatograma (s) de RP-HPLC ✓ Espectro (s) de masas ✓

Purificación: ✓ MM: 2986,60 Da

Fracciones puras mezcladas: ✓

Observaciones: Se obtuvieron 100 mg de péptido con 98,58 % de pureza

Operario: Lazara C. Guerra Firma: [illegible]

Cargo: Analista Fecha: 15/02/2024

Supervisado por: David Diago Alvarez Firma: [illegible]

Cargo: J' Lab Anal- Purif. Fecha: 19/02/2024

Recibido por: Anabel Serrano Díaz Firma: [illegible]

Fecha: 19/02/2024

DOCUMENTO VIGENTE

Supplementary Figure S2: Certificate of CIGB-258 analysis.

Supplementary Figure S3

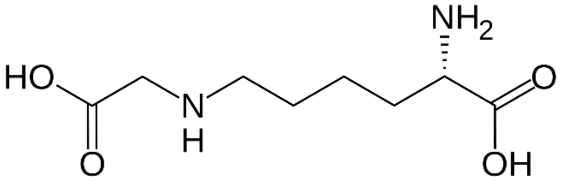

Supplementary Figure S3: Structure of carboxymethyllysine (CML). Molecular formula: $C_8H_{16}N_2O_4$; Molecular mass: 204.226 g·mol^{-1}.

Supplementary Figure S4

Original certificate of water quality

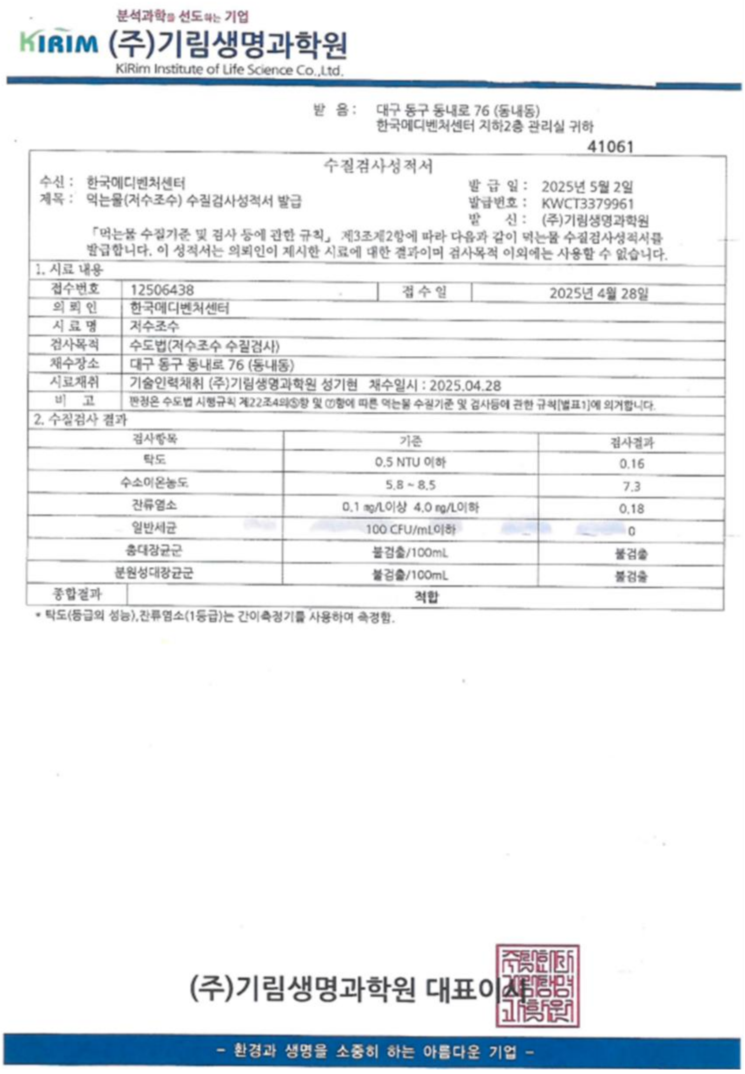

분석과학을 선도하는 기업

KIRIM (주)기림생명과학원

KiRim Institute of Life Science Co.,Ltd.

받 음: 대구 동구 동내로 76 (동내동)
한국메디벤처센터 지하2층 관리실 귀하

41061

수질검사성적서

수신 : 한국메디벤처센터
제목 : 먹는물(저수조수) 수질검사성적서 발급

발 급 일 : 2025년 5월 2일
발급번호 : KWCT3379961
발 신 : (주)기림생명과학원

「먹는물 수질기준 및 검사 등에 관한 규칙」 제3조제2항에 따라 다음과 같이 먹는물 수질검사성적서를 발급합니다. 이 성적서는 의뢰인이 제시한 시료에 대한 결과이며 검사목적 이외에는 사용할 수 없습니다.

1. 시료 내용

접수번호	12506438	접 수 일	2025년 4월 28일
의 뢰 인	한국메디벤처센터		
시 료 명	저수조수		
검사목적	수도법(저수조수 수질검사)		
채수장소	대구 동구 동내로 76 (동내동)		
시료채취	기술인력채취 (주)기림생명과학원 성기현 채수일시 : 2025.04.28		
비 고	판정은 수도법 시행규칙 제22조4의⑤항 및 ⑦항에 따른 먹는물 수질기준 및 검사등에 관한 규칙[별표1]에 의거합니다.		

2. 수질검사 결과

검사항목	기준	검사결과
탁도	0.5 NTU 이하	0.16
수소이온농도	5.8 ~ 8.5	7.3
잔류염소	0.1 ㎎/L이상 4.0 ㎎/L이하	0.18
일반세균	100 CFU/mL이하	0
총대장균군	불검출/100mL	불검출
분원성대장균군	불검출/100mL	불검출
종합결과	적합	

* 탁도(등급외 성능),잔류염소(1등급)는 간이측정기를 사용하여 측정함.

(주)기림생명과학원 대표이사

- 환경과 생명을 소중히 하는 아름다운 기업 -

Certificate of water quality (translated in English)

Drinking Water Quality Test Report
Recipient: Korea Mediventure Center
B2 Management Office
76 Dongnae-ro, Dong-gu, Daegu (Dongnae-dong), 41061
Issued by: Kirim Life Science Co., Ltd.
Issue Date: May 2, 2025
Report Number: KWCT3379961
Subject: Drinking Water (Reservoir Water) Quality Test Report

Basis of Issuance
This report is issued in accordance with Article 3, Paragraph 2 of the *Regulations on Drinking Water Quality Standards and Testing.*
It presents the results of the sample provided by the client. The report must not be used for purposes other than water quality testing.

1. Sample Information
•**Receipt Number:** 12506438
•**Receipt Date:** April 28, 2025
•**Client:** Korea Mediventure Center
•**Sample Name:** Reservoir Water
•**Purpose of Test:** Waterworks Act (Reservoir Water Quality Test)
•**Sampling Location:** 76 Dongnae-ro, Dong-gu, Daegu (Dongnae-dong)
•**Collected by:** Technical staff (Seong Ki-hyun, Kirim Life Science Co., Ltd.)
•**Sampling Date/Time:** April 28, 2025
•**Remarks:** Evaluation conducted in accordance with Article 22, Paragraph 4 of the Enforcement Rules of the Waterworks Act and Annex 1 of the *Regulations on Drinking Water Quality Standards and Testing.*

2. Test Results

Test Item	Standard	Result
Turbidity	≤ 5 NTU	0.16
pH (Hydrogen Ion)	5.8 – 8.5	7.3
Residual Chlorine	0.1 – 4.0 mg/L	0.18
General Bacteria	≤ 100 CFU/mL	0
Total Coliform Group	Not detected / 100 mL	Not detected
Fecal Coliform Group	Not detected / 100 mL	Not detected

Comprehensive Result: Suitable (Compliant)

Note: Turbidity (non-graded performance) and residual chlorine (Grade 1) were measured using a simplified measuring device.

Certification
Issued by: **Kirim Life Science Co., Ltd.** Representative Director
- A beautiful company that values environment and life -

Supplementary Figure S4: Certificate of water quality analysis.

Section S1.

1. Malondialdehyde (MDA), sulfhydryl group, ferric ion reduction (FRA) activity and paraoxonase (PON) activity

The blood malondialdehyde (MDA) level was quantified by mixing plasma sample (20 μL, equivalent to 1 mg/mL protein) with trichloroacetic acid (50 μL, 0.2 mg/μL, pH 1.4) and thiobarbituric acid (100 μL, 6.7 μg/μL, pH 2.3). Following a 10-min incubation at 95 °C, the absorbance at 560 nm was recorded.

The sulfhydryl group was quantified by mixing 60 μL of plasma (1 mg/mL protein) with 60 μL of 5,5′-dithio-bis-(2-nitrobenzoic acid) (DTNB) (4 mg/mL). After 12 hr incubation at room temperature, absorbance 412 nm was determined, and sulfhydryl groups were quantified utilizing 13,600 M^{-1} cm^{-1} extinction coefficient (ε) of DTNB.

To assess ferric ion reduction (FRA) capacity, 20 μL of the plasma (1 mg/mL equivalent protein) was mixed with 180 μL of FRA reagent (prepared by blending 10 mL of acetate buffer (0.2M, pH 3.6) with 1.25 mL each of 2,4,6-tripridyl-S triazin (10 mM, pH 1.9) and $FeCl_3$ (20 mM, pH 2.1). After incubating the mixture at RT for 60 min, absorbance was measured at 593 nm. The results were quantified in μM ferric equivalents based on a ferrous sulfate standard curve.

For paraoxonase activity 20 μL of plasma (1 mg/mL equivalent protein) was mixed with 180 μL of buffer (pH 8.3) [Tris-HCl (90 mM), NaCl (3.6 mM), $CaCl_2$ (90 mM)] containing the paraoxon-ethyl substrate (0.55 M). After 60 min incubation at 25°C, an absorbance (415 nm) was recorded using Microplate reader (Model, iMark™ S.N 21275, Bio-Rad, Hercules, CA, USA) to quantify the production of *p*-nitrophenol, a hydrolysis product of paraoxon-ethyl. Results are expressed as μU/L/min employing the extinction coefficient 17,000 $M^{-1}cm^{-1}$ for *p*-nitrophenol.

Section S2.

2. Method to quantify plasma levels of total cholesterol (TC), triglycerides (TGs), high-density lipoprotein cholesterol (HDL-C) and hepatic function biomarkers aspartate transaminase (AST) and alanine transaminase (ALT)

The plasma total cholesterol (TC) and triglycerides (TGs) were determined using commercial assay kits (cholesterol, AM 202-K, and TGs, AM 157-K, Asan Pharmaceutical, Hwasung, Republic of Korea) as per the method suggested by the suppliers. In brief, 5 μL serum was mixed with 200 μL reaction mixture (supplied with a commercial assay kit) for the TC analysis. The content was incubated at 37°C for 10 min, resulting in a red-colored product quantified by adsorption at 490 nm (Microplate reader, iMark™ Bio-Rad, Hercules, CA, USA).

Similarly, 5 μL serum was mixed with a 200 μL of TGs-specific reaction mixture (supplied with a commercial assay kit) for TGs analysis. The content was incubated for 10 min at 37°C, and the formed colored product was quantified by taking adsorption at 490 nm.

For HDL-C analysis, serum was mixed in an equal ratio with the separation solution (supplied with a commercial assay kit), followed by centrifugation at 3,000 rpm for 10 min at 25°C. The supernatant (20 μL) was collected and blended with a 200 μL reaction mixture (supplied with a commercial assay kit). After 10 min incubation at 37°C, red color intensity corresponding to HDL-C was quantified by taking absorption at 490 nm (Microplate reader, iMark™ Bio-Rad, Hercules, CA, USA).

The commercial diagnostic kit (AM102K and AM103-K, Asan Pharmaceutical, Hwasung, Republic of Korea) was used to quantify aspartate transaminase (AST) and alanine transaminase (ALT) levels in the plasma, following the instructions suggested by the manufacturers. Briefly, 5 μL of plasma

was combined with 250 μL of either AST or ALT-specific solution, as supplied in the diagnostic kit. Following a 30 min incubation for AST or 60 min incubation of ALT at 37°C, the mixture was then blended with 250 μL of the respective coloring reagent (AST or ATL-specific, provided in the diagnostic kit). After a subsequent 20 min incubation at room temperature, 250 μL of 0.4 N NaOH was introduced to halt the reaction. Finally, the AST and ATL were quantified by measuring absorbance at 490 nm (Microplate reader, iMark™, Bio-Rad, Hercules, CA, USA).
